# Reference transcriptomes and comparative analyses of six species in the threatened rosewood genus *Dalbergia*

**DOI:** 10.1038/s41598-020-74814-2

**Published:** 2020-10-20

**Authors:** Tin Hang Hung, Thea So, Syneath Sreng, Bansa Thammavong, Chaloun Boounithiphonh, David H. Boshier, John J. MacKay

**Affiliations:** 1 Department of Plant Sciences, University of Oxford, Oxford, OX1 3RB UK; 2 Institute of Forest and Wildlife Research and Development, Phnom Penh, Cambodia; 3 Forest Research Center, National Agriculture and Forestry Research Institute, Vientiane, Lao PDR

**Keywords:** Conservation biology, Genetic markers, Sequencing

## Abstract

*Dalbergia* is a pantropical genus with more than 250 species, many of which are highly threatened due to overexploitation for their rosewood timber, along with general deforestation. Many *Dalbergia* species have received international attention for conservation, but the lack of genomic resources for *Dalbergia* hinders evolutionary studies and conservation applications, which are important for adaptive management. This study produced the first reference transcriptomes for 6 *Dalbergia* species with different geographical origins and predicted ~ 32 to 49 K unique genes. We showed the utility of these transcriptomes by phylogenomic analyses with other Fabaceae species, estimating the divergence time of extant *Dalbergia* species to ~ 14.78 MYA. We detected over-representation in 13 Pfam terms including HSP, ALDH and ubiquitin families in *Dalbergia*. We also compared the gene families of geographically co-occurring *D. cochinchinensis* and *D. oliveri* and observed that more genes underwent positive selection and there were more diverged disease resistance proteins in the more widely distributed *D. oliveri*, consistent with reports that it occupies a wider ecological niche and has higher genetic diversity. We anticipate that the reference transcriptomes will facilitate future population genomics and gene-environment association studies on *Dalbergia*, as well as contributing to the genomic database where plants, particularly threatened ones, are currently underrepresented.

## Introduction

The genus *Dalbergia* Linn. f. (Fabaceae: Faboideae) contains around 250 species, many of which are globally recognized for their economic value. *Dalbergia* species encompass a high diversity in their life histories and morphologies as trees, shrubs, and woody lianas^1^. They are distributed pantropically across Central and South Americas, Africa, and Asia^2^. More than 50 *Dalbergia* species are documented to have the ability to fix atmospheric nitrogen with possession of aeschynomenoid type root nodules^3^. Many *Dalbergia* species produce valuable heartwood timber known as rosewood, and are incorporated in a wide range of uses including furniture, boats, and musical instruments^4^. They are often targeted in illegal harvesting and traded in local and global markets with little regulation either in Asia (including the Indochina biodiversity hotspot) or Africa (particularly in Madagascar)^5,6^. Due to overexploitation of their timber, population sizes and areas within their native distribution have significantly diminished^7^. The genus *Dalbergia* is declared as threatened worldwide, with many species classified as endangered or vulnerable in the International Union for Conservation of Nature (IUCN) Red List. The whole genus of *Dalbergia* was listed in the Convention on International Trade in Endangered Species (CITES) Appendix I or II in 2017 to regulate the international trade of *Dalbergia* timber.

Studies in the evolutionary history and genetic resources of *Dalbergia* are still scarce. Genetic markers have been developed for a number of *Dalbergia* species and used in studies of evolutionary history and for conservation. The earliest complete report on infrageneric taxonomy of *Dalbergia* was published by Bentham^8^, and the first molecular phylogeny recently supported the monophyletic nature of *Dalbergia* genus, grouped in a clade with other genera including *Machaerium, Aeschynomene*, and *Ormocarpum*^1^. In earlier studies the *Dalbergia* clade was assigned to the Dalbergieae tribe with *Adesmia* and *Pterocarpus* clades^9^. Recent studies utilise genetic markers to infer the phylogeography of populations and identify landscape features which may explain the population structure^10^. A number of DNA-based barcodes have also been developed that may be used in conservation forensics to track illegal trade and verify species identification^11^. These *Dalbergia* studies have mainly analysed loci such as *rbcL*, *matK*^4,12^, *trnL*, and *psbA-trnH*^13^ at species level, and microsatellites^10,14,15^ at the infraspecific level. Although recent advances in high-throughput sequencing have expanded the assembly repertoire of many species, genomic resources for the genus *Dalbergia* remain scarce for such a big genus: namely one de novo transcriptome assembly of *D. odorifera*^16^ (without a gene annotation report), and ten chloroplast genomes^17–21^.

The genomic resource gap potentially hinders the understanding of evolutionary history in *Dalbergia* and the application of genetic tools in conservation. For example, *D. cochinchinensis* and *D. oliveri* are commonly found in the same geographical localities in South Eastern Asia, but they have significantly different neutral genetic structure^10^. Understanding their adaptive differences using genome-wide analyses would help devise potentially different conservation strategies. Due to the lack of a reference genome for any of the *Dalbergia* species, transcriptomes can be a practical starting point to facilitate evolutionary research and conservation applications. High-throughput sequencing technologies for RNA-seq enable gene prediction and annotation for non-model organisms with scarce genomic information^22^.

In this study, we develop a resource and knowledge base to facilitate transferability and utility across the genus. We produced the first reference transcriptomes from de novo assemblies for six diverse *Dalbergia* species, including *D. cochinchinensis* Pierre, *D. frutescens* (Vell.) Britton, *D. melanoxylon* Guill. & Perr., *D. miscolobium* Benth., *D. oliveri* Gamble ex Prain, and *D. sissoo* Roxb. ex DC. (Table 1). For gene annotation, we used ab-initio gene prediction based on the structure of open reading frames, features of protein-coding genes, and sequence homology to gene models of closely related species^23^. To demonstrate the utility of the transcriptomic resources, we conducted phylogenomic, gene enrichment, and selection analyses comparing the *Dalbergia* and other Fabaceae species.

**Table 1:** Basic details and conservation status of the 6 *Dalbergia* species covered in this study.

Scientific name	Common name	Native occurrence	Habitat	IUCN status	CITES status	References
*Dalbergia cochinchinensis*	Siamese rosewood	Cambodia, Lao PDR, Thailand, Vietnam	Terrestrial; open semi-deciduous forests	Vulnerable A1cd (1998)	II (2017)	^105^
*Dalbergia frutescens*	Brazilian tulipwood	Columbia, Amazonia, Andes, Caribbean Plain, Magdalena Valley	Variable, usually as a liana	Unclassified	II (2017)	^106^
*Dalbergia melanoxylon*	African blackwood	Wide geographical distribution in sub-Saharan countries	Range of woodland habitats	Near threatened (1998)	II (2017)	^107^
*Dalbergia miscolobium*	Jacaranda-do-cerrado	Brazil, Bolivia	Savannah	Unclassified	II (2017)	^108^
*Dalbergia oliveri*	Burmese rosewood	Cambodia, Lao PDR, Myanmar, Thailand, Vietnam	Mixed deciduous forests and tropical evergreen	Endangered A1cd (1998)	II (2017)	^109^
*Dalbergia sissoo*	North Indian rosewood; Shisham	Indian Subcontinent	Deciduous forests	Unclassified	II (2017)	^110^

## Methods

### Ethics statement

*Dalbergia cochinchinensis* and *D. oliveri* are listed as vulnerable and endangered in the IUCN Red List respectively (Table 1). All *Dalbergia* species are listed in the CITES Appendix II, albeit their seeds are exempted according to Annotation #15. The seed collections of *D. cochinchinensis* and *D. oliveri* were made by local government authorities with permissions and licences in place.

### Plant materials and sample preparation

Dried seeds of *Dalbergia cochinchinensis*, *D. frutescens*, *D. melanoxylon*, *D. miscolobium*, *D. oliveri*, and *D. sissoo* were obtained from different sources (Supplementary Table 1) and stored at 4 °C until seed germination. The seeds were scarified by placing them in 70 °C distilled water, which was then left to cool to room temperature for 1 h, with the seed soaking in the water for 24 h. The seeds were germinated in 1% agar in a plant growth cabinet MLR-350 (Sanyo, Watford, United Kingdom) at 25 °C and photoperiod 12L/12D. Seedlings were transferred to small pots in a soil-perlite 3:1 (v:v) mixture in the same growth cabinet. The plants were watered to pot capacity, with any moulded or diseased plants immediately removed. After plant height reached a minimum of 10 cm, four plants of each species were randomly selected. Two plants were drought-stressed until soil gravimetric water content dropped below 50%, while the other two were watered as usual. Three tissues (foliage, stem, and root) were harvested from each individual and their total RNA extracted (*n* = 72) with Monarch Total RNA Miniprep Kit (New England BioLabs, United Kingdom). Multiple tissue types and growth conditions increased the diversity of transcripts towards a more-complete transcriptome^24^. The quantity and quality of total RNA from each sample were determined with NanoDrop 2000 (Thermo, Wilmington, United States). RNA integrity was assessed with the RNA 6000 Nano Assay on a 2100 Bioanalyzer (Agilent Technologies, Santa Clara, United States) and RNA samples with a minimum RNA integrity number (RIN) of 7 (for leaf tissues) and 8 (for root and stem tissues) were retained for RNA-Seq. Samples of the same species were pooled to equimolarity.

### Library preparation and sequencing

RNA samples (*n* = 6) were sent to the Oxford Genomics Centre (Oxford, United Kingdom) for library preparation and sequencing. Polyadenylated transcript enrichment was performed with NEBNext Poly(A) mRNA Magnetic Isolation Module (New England BioLabs), and then individual libraries were prepared with NEBNext Ultra II Directional RNA Library Prep Kit (New England BioLabs). Libraries were amplified on a Tetrad (Bio-Rad) using in-house unique dual indexing primers^25^. Individual libraries were normalised and their size profiles were analysed on the 2200 or 4200 TapeStation (Agilent, RNA ScreenTape). The pooled library was diluted to ~ 10 nM for storage. The 10 nM library was denatured and further diluted prior to loading on the sequencer. Paired-end sequencing was performed on the HiSeq4000 (Illumina, HiSeq3000/4000 PE Cluster Kit and 150 cycle SBS Kit) with a read length of 150 bp. The raw reads were obtained in fastq files after an in-house preliminary quality check.

### Data filtering and de novo assembly

**Figure 1:**
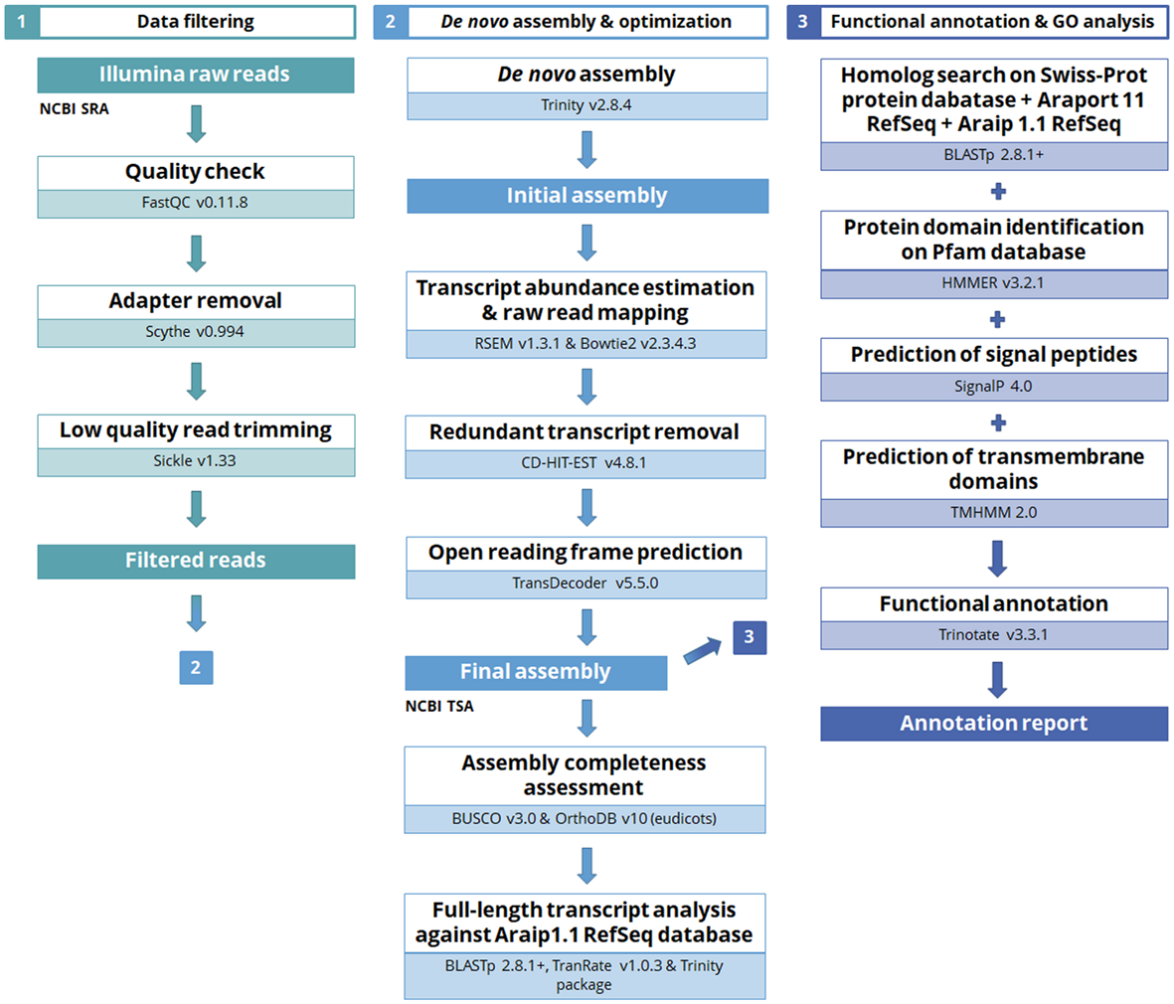
Bioinformatic pipeline of de novo transcriptome analysis and gene annotation for the 6 *Dalbergia* species. For the software details, see “Methods”.

Quality of raw reads was examined using FastQC v0.11.8 and visualized in MultiQC v1.7^26^. Scythe v0.994^27^ was used to trim the 3′-end adapter contaminants and Sickle v1.33^28^ was used to remove the low-quality reads (Phred quality score < 30). Filtered reads were assessed again with FastQC. As no reference genome was available for the genus *Dalbergia*, we assembled the transcriptomes de novo, to avoid the bias that may be introduced by using other species in genome-guided assembly^29^. The filtered reads for each species were de novo assembled using Trinity v2.8.4^30^ with the default parameters. The assembly and subsequent steps were performed on the University of Oxford Advanced Researching Computing ARCUS-B cluster. The schematic bioinformatic pipeline of the transcriptome assembly is shown in Fig. 1.

### Assembly quality assessment and optimization

As a first quality assessment, we generated the output statistics of the initial individual de novo assemblies with Trinity scripts. We then assessed the read content of the transcriptome assembly for each species by mapping the clean reads to the assembly using Bowtie2 v.2.3.4.3^31^ with the options “-p 10 -q --no-unal -k 20”, as suggested in the Trinity package.

Optimizations were carried out to improve the performance and accuracy of downstream analyses, as de novo assembly often produces highly similar transcript sequences such as isoforms or assembly artefacts. First, we reduced the redundancy of transcripts with CD-HIT-EST v4.8.1^32^ by removing transcripts with sequence similarity greater than 95%. Then we estimated candidate coding regions within transcript sequences with TransDecoder v5.5.0^33^ to identify the single best predicted open reading frames (ORF) that are at least 100 amino acids long (parameter--single_best_only). Each transcript was represented by the longest translated protein sequence and each gene by the longest transcript in the final assembly.

We compared the transcripts in the final assembly against the OrthoDB v10 eudicotyledons database^34^ with BUSCO (Benchmarking Universal Single-Copy Orthologs) v3.0^35^ to evaluate the assembly completeness. For full-length transcript analysis, we performed BLASTP searches (--evalue 1e-3) on the non-redundant transcripts against the RefSeq protein data of *Arachis ipaensis* (NCBI: GCF_000816755.2 Araip1.1^36^), which represented the closest relative to *Dalbergia* with an available annotated genome^36^. We then calculated the coverage of aligned transcripts based on their BLAST hits with ‘analyze_blastPlus_topHit_coverage.pl’ script in the Trinity package. We also used TransRate v1.0.3^37^ to obtain the Conditional Reciprocal Best BLAST (CRBB) and coverage metrics of final assemblies using Araip1.1 as a reference.

### Structural and functional annotation

We aligned our final assemblies against the SwissProt database^38^, Araip1.1^36^, and the *Arabidopsis thaliana* database (Araport11)^39^ with BLASTP for best hits with an e-value below the threshold 10^−3^. We then annotated the protein domains with HMMER v3.2.1 (https://hmmer.org) on the Pfam 32.0 database (version September 2018, 17,929 entries)^40^. We also predicted signal peptides using SignalP 5.0^41^ and transmembrane domains using TMHMM 2.0^42^. We finally loaded the blast homologies of three databases (SwissProt, Araport11, and Araip1.1) into an SQLite database and generated the annotation report for each species assembly with Trinotate v3.3.1. GO (Gene Ontology), KEGG (Kyoto Encyclopedia of Genes and Genomes), and COG (Clusters of Orthologous Groups) assignments were transferred from SwissProt annotations as a verified source.

### Phylogenomic analysis and estimation of divergence time

We ran OrthoFinder v2.2^43^ on the 6 *Dalbergia* transcriptomes in this study and 10 other Fabaceae species (Supplementary Table 2). After the analysis, only single-copy orthologs among taxa were retrieved as they were the most robust for phylogenetic reconstruction with high confidence and concordance^44^. We performed multiple sequence alignment for each set of single-copy orthologs using MAFFT v7^45^, and every corresponding coding sequence was retrieved and matched to ortholog alignment with PAL2NAL v14^46^. Coding sequences of all ortholog alignments were concatenated to create a single multiple sequence alignment (https://github.com/nylander/catfasta2phyml).

**Table 2:** Summary of transcriptome assembly statistics of the 6 *Dalbergia* species.

Feature	*D. cochinchinensis*	*D. frutescens*	*D. melanoxylon*	*D. miscolobium*	*D. oliveri*	*D. sissoo*
Number of paired-end raw reads	168,351,690	71,187,798	74,366,734	91,273,654	181,456,683	73,160,910
Number of paired-end filtered reads	156,116,637 (92.7%)	65,092,217 (91.4%)	67,994,105 (91.4%)	83,178,635 (91.1%)	169,551,748 (93.4%)	67,086,967 (91.7%)
Number of transcripts in initial assembly	277,981	274,663	363,116	208,249	376,014	195,268
Number of genes in initial assembly	161,051	179,085	212,141	123,962	223,289	121,629
Total length of transcripts (bp)	316,346,363	255,266,594	309,909,355	237,557,440	357,336,705	216,910,975
Average transcript length (bp)	1,138.01	929.38	853.47	1,140.74	950.33	1,110.84
N50^1^ (bp)	2,159	1,749	1,477	2,074	1,851	2,019
GC (%)	40.25	41.88	41.38	40.60	40.59	41.06
Map representation alignment rate (%)	89.85	87.71	85.69	89.20	87.14	89.02
Number of non-redundant transcripts	224,511	231,281	271,088	174,382	293,334	168,039
**Final assembly**						
Number of transcripts in final assembly	84,003	84,897	80,484	69,357	92,906	67,379
Total length of transcripts (bp)	81,157,122	75,431,325	70,467,927	68,915,367	83,501,667	67,138,149
Average transcript length (bp)	966.12	888.50	875.55	993.63	898.78	996.43
N50 of transcripts (bp)	1,254	1,152	1,149	1,290	1,179	1,305
GC of transcripts (%)	44.66	46.01	45.24	44.68	44.97	45.00
Number of genes in final assembly	34,655	48,591	43,848	31,678	43,879	32,753
Total length of genes (bp)	33,219,183	41,338,207	37,309,763	31,488,922	37,371,154	32,374,118
Average gene length (bp)	958.57	850.74	850.89	994.03	851.69	988.43
N50 of genes (bp)	1,341	1,145	1,173	1,383	1,182	1,374
GC of genes (%)	45.37	47.43	46.00	45.32	45.97	45.92
BUSCO Score^2^ (N = 2,121) (%)	C: 92.2; F: 4.9; M: 2.9	C: 92.1; F: 4.8; M: 3.1	C: 92.3; F: 5.1; M: 2.6	C: 93.1; F: 4.6; M: 2.3	C: 90.9; F: 6.5; M: 2.6	C: 94.4; F: 3.3; M: 2.3

^1^Sequence length of the shortest contig at 50% of the total transcriptome length.

^2^Results of BUSCO analysis; (%) per category: C: complete, F: fragmented, M: missing, N: number of BUSCOs tested in the OrthoDB v10 eudicot dataset.

The nucleotide substitution model was tested on the concatenated alignment with jModelTest 2.1.10^47^ for likelihood scores. The alignment was then used to construct a best-fit (i.e. GTR + Γ + I) maximum likelihood phylogenetic tree using RAxML (Randomized Axelerated Maximum Likelihood) 8.2.12^48^ with 100 rapid bootstrapping. The maximum likelihood (ML) tree was used as a starting tree in both the Bayesian phylogenetic analysis and subsequently in the gene family analysis.

We estimated the species divergence time with BEAST (Bayesian evolutionary analysis by sampling trees) v.2.5.2^49^ using a calibrated birth-death model with an uncorrelated lognormal relaxed clock (ULRC). The crown age of the tree (Fabaceae) was calibrated to the oldest definitive legume fossil (wood of *Paracacioxylon frenguellii*) at 63.5 million years ago (MYA)^50^. The crown age of Faboideae was calibrated to 56.3 ± 1.05 MYA^51^ and that of the Dalbergoid clade (*Nissolia*-*Dalbergia* split) was calibrated to 50.7 ± 0.8 MYA^52^. The time of the *A. duranensis-A. ipaensis* split was calibrated to 2.88 ± 0.22 MYA^53^. All nodes were calibrated to normal models and their sigma values estimated a priori. We ran 15,000,000 iterations with 150,000 burn-ins for the Monte Carlo Markov chain and also ran 15,000 trees with 10% burn-ins to produce the maximum clade credibility tree.

### Enrichment analysis and gene family evolution

*Acrocarpus fraxinifolius*, *Bauhinia tomentosa*, and *Xanthocercis zambesiaca* were excluded from the subsequent Pfam and CAFE (Computational Analysis of gene Family Evolution) analyses as their BUSCO scores were not reported in their original studies, and incomplete transcriptomes could introduce bias to the enrichment and gene family analyses.

Gene annotations of the *Dalbergia* species from the Trinotate pipeline were subject to enrichment analyses. First, the annotated GO terms were extracted and searched against the WEGO (Web Gene Ontology Annotation Plot) 2.0 database^54^ (version 1 November 2018) to count the level-2 GO terms for each of the *Dalbergia* species. A chi-square test of independence was conducted to detect under- and over-represented GO terms among the species and significant terms were presented in chord diagrams (https://github.com/mattflor/chorddiag). Second, the annotated Pfam domains were extracted for each species and under- and over-represented Pfam terms were determined using a two-tailed Fisher’s exact test. The mean Pfam domain counts in *Dalbergia* were compared against the background domain counts of the other Fabaceae species. Row-Z scores for each significant Pfam domain were used to construct a heatmap in R version 3.6.3.

We applied CAFE version 3.1^55^ based on a Bayesian method to detect gene family contraction/expansion events, where a gene family is defined as the orthogroup clustered in the previous OrthoFinder pipelines. We used the ultrametric tree resulting from the Bayesian phylogenetic analysis to time-calibrate the gene trees. For each orthogroup we computed the family-wide *p* value and branch-specific *p* value (using the Viterbi method) to test the significance of a contraction/expansion event at a specific branch. As recommended by the software developers, only orthogroups with a family-wide *p* value < 0.05 and a branch Viterbi *p* value < 0.001 were considered significant. We then used PANTHER version 15.0^56^ to detect significant over-/under-represented GO terms (*p* < 0.05 after Benjamini and Hochberg correction) of biological functions in the significantly expanded gene families after CAFE analysis.

### Positive selection analysis

Single-copy orthologs of the 6 *Dalbergia* species were extracted using the Orthofinder pipeline. The rooted trees for each set of orthologs obtained from RAxML were used to support the evolutionary relationship of the species, while gene signatures of positive selection along a specific branch were detected by branch-site models in the codeml function of PAML (Phylogenetic Analysis by Maximum Likelihood) 4.9^57^. We set *D. cochinchinensis* and *D. oliveri*, which show overlapping ranges in South Eastern Asia^10^, as the foreground phylogeny and other species as the background phylogeny in the branch-site model. We built the alternative model (i.e. the foreground phylogeny has genes under positive selection) for each ortholog with the codeml setting: model = 2, NSites = 2, fix_kappa = 0, fix_omega = 0, omega = 1; and the null model (i.e. the foreground phylogeny has genes under neutral selection compared to the background phylogeny) with the codeml setting: model = 2, NSsites = 2, fix_kappa = 0, fix_omega = 1 and omega = 1. Sites under positive selection were defined as those with higher nonsynonymous-to-synonymous substitution ratios (*d*_*N*_*/d*_*S*_) > 1, as expected under neutral evolution. The two hypothetical models were tested for likelihood ratio using a chi-squared distribution with one degree of freedom, following the Benjamini and Hochberg method to correct for the significance level^58^. We determined the positively selected genes as those with corrected *p* < 0.1^59^. KEGG pathway and module enrichment tests were performed on positively selected genes using enrichKEGG and enrichMKEGG functions in clusterProfiler v3.0.4^60^ respectively, with *Arachis ipaensis* set as the reference organism.

## Results

### RNA-seq library construction and sequencing

Total RNA was successfully extracted from leaf, stem and root tissues of each of 6 *Dalbergia* species and the RNA integrity numbers (RIN) of the RNA pools were all above 7.0. HiSeq4000 multiplex sequencing yielded between 71 to 180 million paired end reads of 150 bp length for each of the 6 *Dalbergia* species (Table 2). After quality filtering and trimming, more than 90% of the reads were retained with quality scores ≥ 30. The raw read data from Illumina sequencing for each species are deposited in the NCBI Sequence Read Archive (SRR: SRR10592611–SRR10592618) under BioProject PRJNA593817.

### De novo transcriptome assembly and transcript filtering

The number of transcripts in initial de novo assemblies from Trinity ranged between 195,268 and 376,014 (see Table 2 for assembly statistics). As the first step of assembly quality assessment, we successfully mapped 86–90% of the raw filtered reads to individual assemblies, where an alignment rate above 80% indicates a good quality assembly^30^.

Redundant transcripts were identified by clustering similar transcripts and open reading frame prediction to produce the final assemblies (Fig. 1), which filtered roughly 65–75% of the transcripts. In the final assemblies, 67,379–92,906 transcripts were captured for individual species, and predicted to correspond to 31,678–48,591 unique genes. The final assemblies are deposited in the NCBI Transcript Shotgun Archive (GIHP00000000–GIHU00000000).

The BUSCO procedure confirmed that the majority of eudicot core genes were captured in our transcriptomes indicating high completeness of our transcriptome assemblies. Search for the 2121 orthologs recovered over 90% of complete BUSCOs in all of our assemblies with fewer than 5% of BUSCOs missing (Supplementary Table 3).

**Table 3:** Transcriptome annotation statistics of the 6 *Dalbergia* species. For the versions of annotation databases, see “Methods” for details.

	*D. cochinchinensis*	*D. frutescens*	*D. melanoxylon*	*D. miscolobium*	*D. oliveri*	*D. sissoo*
Number of transcripts in final assembly	84,003	84,897	80,484	69,357	**92,906**	67,379
**Number of successfully annotated TRANSCRIPTS**						
Araip 1.1	74,397 (88.6%)	67,052 (79.0%)	67,164 (83.5%)	**61,653 (88.9%)**	78,245 (84.2%)	58,512 (86.8%)
Araport 11	70,780 (84.3%)	63,438 (74.7%)	62,185 (77.3%)	**58,984 (85.0%)**	73,889 (79.5%)	56,091 (83.2%)
SwissProt	63,175 (75.2%)	61,062 (71.9%)	56,193 (69.8%)	**53,201 (76.7%)**	67,064 (72.2%)	51,051 (75.8%)
GO	61,993 (73.8%)	60,005 (70.7%)	55,022 (68.4%)	**52,043 (75.0%)**	65,740 (70.8%)	50,008 (74.2%)
KEGG	55,538 (66.1%)	52,709 (62.1%)	48,603 (60.4%)	**46,789 (67.5%)**	57,896 (62.3%)	45,190 (67.1%)
EggNOG	52,510 (62.5%)	44,849 (52.8%)	44,802 (55.7%)	**44,221 (63.8%)**	54,059 (58.2%)	41,184 (61.1%)
Pfam	58,589 (69.7%)	56,835 (66.9%)	51,717 (64.3%)	**49,888 (71.9%)**	62,162 (66.9%)	47,842 (71.0%)
TMHMM	**17,486 (20.8%)**	15,424 (18.2%)	14,864 (18.5%)	14,338 (20.7%)	18,359 (19.8%)	13,671 (20.3%)
SignalP	5603 (6.7%)	5214 (6.1%)	4880 (6.1%)	**4772 (6.9%)**	5896 (6.3%)	4643 (6.9%)
Number of genes in final assembly	34,655	**48,591**	43,848	31,678	43,879	32,753
**Number of successfully annotated GENES**						
Araip 1.1	28,277 (81.6%)	33,452 (68.8%)	33,617 (76.7%)	**26,315 (83.1%)**	32,936 (75.1%)	26,141 (79.8%)
Araport 11	26,388 (76.1%)	31,421 (64.7%)	30,420 (69.4%)	**24,894 (78.6%)**	30,497 (69.5%)	24,837 (75.8%)
SwissProt	24,175 (69.8%)	32,281 (66.4%)	28,022 (63.9%)	**22,920 (72.4%)**	28,658 (65.3%)	23,396 (71.4%)
GO	23,686 (68.4%)	31,733 (65.3%)	27,471 (62.7%)	**22,427 (70.8%)**	28,116 (64.1%)	22,926 (70.0%)
KEGG	20,603 (59.5%)	27,102 (55.8%)	23,609 (53.8%)	19,606 (61.9%)	23,810 (54.3%)	**20,297 (62.0%)**
EggNOG	19,163 (55.3%)	20,886 (43.0%)	21,121 (48.2%)	**18,204 (57.5%)**	21,470 (48.9%)	17,635 (53.8%)
Pfam	23,134 (66.8%)	30,561 (62.9%)	26,161 (59.7%)	**22,077 (69.7%)**	27,332 (62.3%)	22,544 (68.8%)
TMHMM	**7609 (22.0%)**	8120 (16.7%)	7748 (17.7%)	6417 (20.3%)	8006 (18.3%)	6447 (19.7%)
SignalP	2607 (7.5%)	3060 (6.3%)	2763 (6.3%)	**2453 (7.7%)**	2874 (6.6%)	2401 (7.3%)

Highest numbers for each row are highlighted in bold.

We mapped our transcripts to gene models of *Arachis ipaensis*, with near full-length and fragmented transcripts defined as > 70% and < 30% coverage respectively. We found that roughly 80% of the transcripts were near full-length for all transcriptomes, with only 5–8% of fragmented transcripts (Supplementary Fig. 1). There was no evidence for mapping bias among the species when comparing the counts of full-length and fragmented transcripts among our transcriptomes (*p* > 0.05, chi-square test of independence). The TransRate analysis returned a high mean percentage of contigs covered by the ORF (> 99.7% for all assemblies) and a rather low coverage on the *A. ipaensis* reference (~ 34.1% for all assemblies) (Supplementary Table 4). However, the reference coverage depends significantly on the evolutionary distance between the assembled and reference species^37^.

### Structural and functional annotation

We annotated the *Dalbergia* transcriptome assemblies by using multiple sources and methods to provide a complete set of annotations for each species. We separated the annotations for our full transcriptome assemblies, which contained isoforms from alternative splicing as predicted in the Trinity pipeline and the gene set, which only contained the longest isoform representing each gene. The homology search on *Arachis ipaensis*, *Arabidopsis thaliana*, and SwissProt annotated 69.8–88.9% of the transcripts and 63.9–83.1% of the genes, depending on the *Dalbergia* species. We also identified protein domains (as Pfam terms) on 59.8–69.8% of the genes, transmembrane domains on 16.7–20.2% of the genes, and signal peptides on 6.3–7.7% of the genes. GO, KEGG and EggNOG assignments were transferred from SwissProt/UniProtKB annotations. The annotation report for each species assembly is available (Supplementary Data 1), and the annotation statistics for the transcriptomes are shown in Table 3.

### Phylogenomic analysis and estimation of divergence time

**Figure 2:**
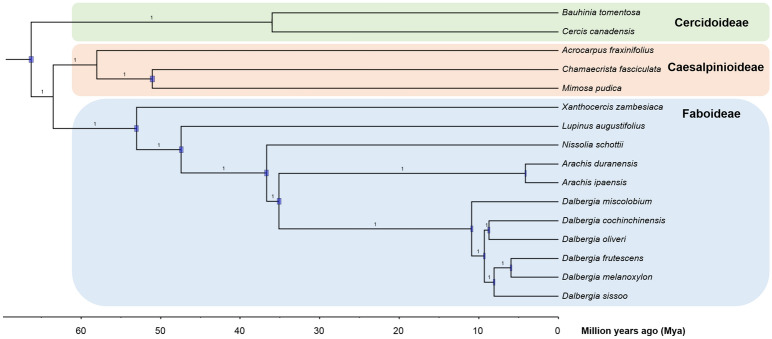
Dated phylogeny of 16 Fabaceae species based on Bayesian analysis of a supergene from the 256 single-copy orthologs (479,064 bp) from their transcriptomes. Node bars indicate 95% CI for the estimated divergence time. Numbers on branches indicate posterior probability (1 for all branches).

Analysis using Orthofinder assigned 481,614 genes (84.7% of total genes) in our 6 *Dalbergia* and 10 other Fabaceae transcriptomes into 34,725 orthogroups (Supplementary Table 5). All species present shared 5493 orthogroups but only 256 orthogroups contained single-copy genes. The *Dalbergia* species shared 13,149 orthogroups (Supplementary Fig. 2). A Bayesian phylogenetic tree constructed using these 256 single-copy orthologs, with a total aligned length of 479,064 bp, supported the monophyly of *Dalbergia* species in the present study and showed the expected relationship of *Dalbergia* species with other major Fabaceae groups (Fig. 2).

Using the multiple fossil calibration nodes in Fabaceae, we estimated the divergence time of extant members of the genus *Dalbergia* to be around 14.78 MYA (95% HPD: 13.74 – 16.02). The divergence times of other branches are shown in Supplementary Table 6.

### Enrichment analyses and gene family evolution

GO enrichment analyses revealed significant differences for GO categories of cellular components, biological processes, and molecular functions among *Dalbergia* species (Supplementary Table 7 and Supplementary Fig. 3; *p* < 0.05, chi-square test of independence). In most categories, *D. frutescens* and *D. oliveri* had the most GO term counts, whereas *D. miscolobium* and *D. sissoo* had the fewest counts. The pattern of GO term count reflected the number of genes predicted in the assemblies, where *D. frutescens* had the highest number of genes (49,050) and *D. miscolobium* the lowest (32,107).

**Figure 3:**
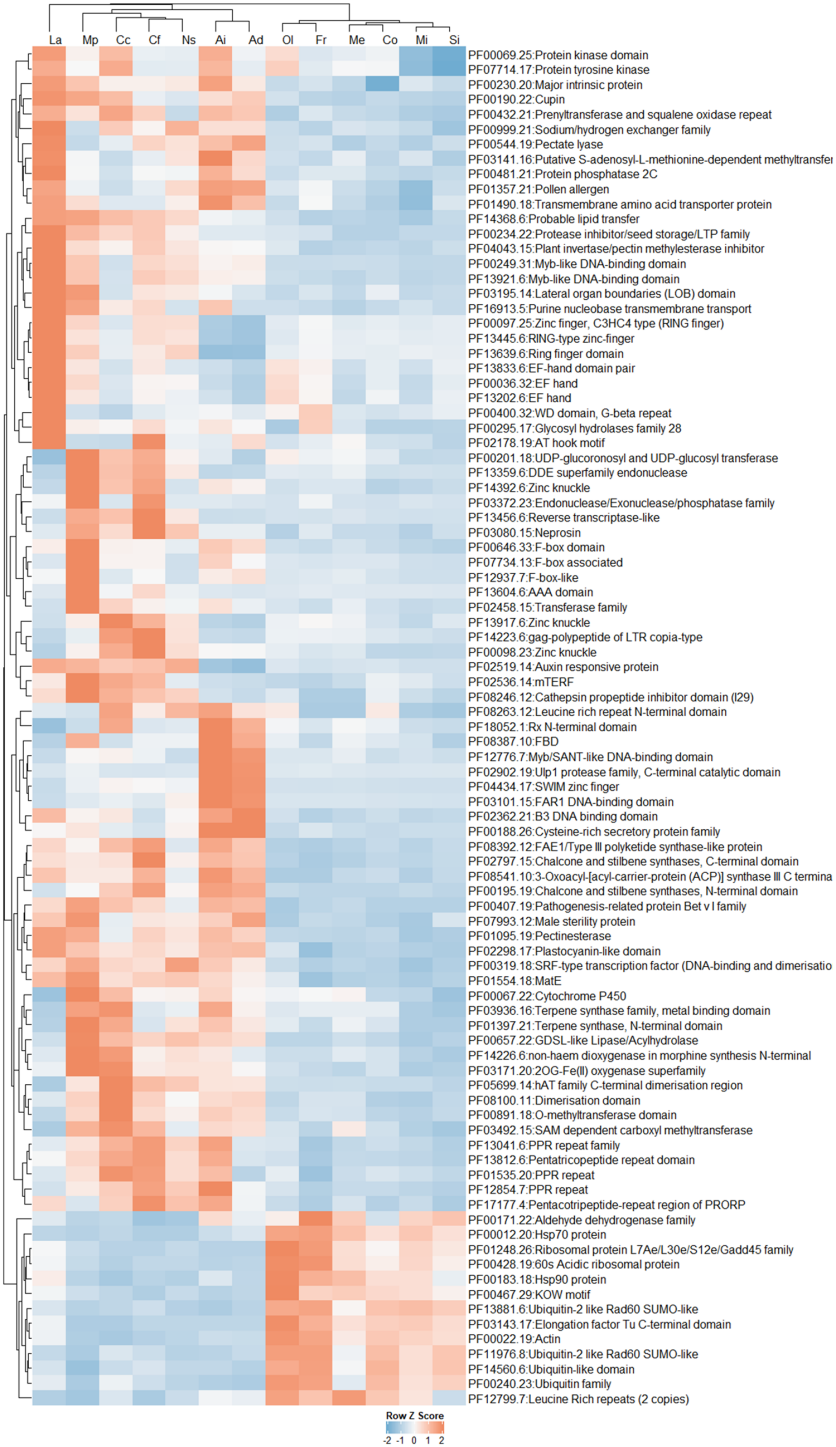
Heatmap of annotated Pfam domains of the 13 Fabaceae species, only showing domains (*n* = 91) that are significantly contracted (negative) or expanded (positive) in the *Dalbergia* species (*p* < 0.05, two-tailed Fisher’s exact test of independence). See Supplementary Table 2 for species abbreviations.

We conducted enrichment analyses on the Pfam protein domains to determine over- or under-represented specific groups of genes between *Dalbergia* species and other Fabaceae species (Supplementary Table 8 and Fig. 3; *p* < 0.05, two-tailed Fisher’s exact test). While we reported a list of under-represented protein domains in *Dalbergia* species, we were cautious about the completeness of our transcriptome assemblies, owing to the samples only including juvenile stage vegetative tissues. We focused on the 13 protein domains that were over-represented in our *Dalbergia* study species. These included two heat shock proteins Hsp70 and Hsp90 (PF00012.20 and PF00183.18), ubiquitin-related proteins (PF13881.6, PF11976.8, PF14560.7, and PF00240.23), aldehyde dehydrogenase family (PF00171.22), ribosomal proteins (PF01248.26 and PF00428.19), KOW motif (PF00467.29), elongation factor (PF03143.17), actin (PF00022.19), and leucine rich repeats (PF12799.7).

To detect the local scale of gene family expansion/contraction events in *D. cochinchinensis* and *D. oliveri*, CAFE analysis revealed 10 and 49 orthogroups that significantly expanded respectively (family-wide *p* value < 0.05, branch Viterbi *p* value < 0.001; Supplementary Table 9. GO enrichment analysis revealed many over-represented terms (BH *p* < 0.05, two-tailed Fisher’s exact test; Supplementary Table 10) in these significantly expanded gene families, including innate immune response (GO:0045087) and defence response (GO:0006952).

### Positive selection analysis

**Figure 4:**
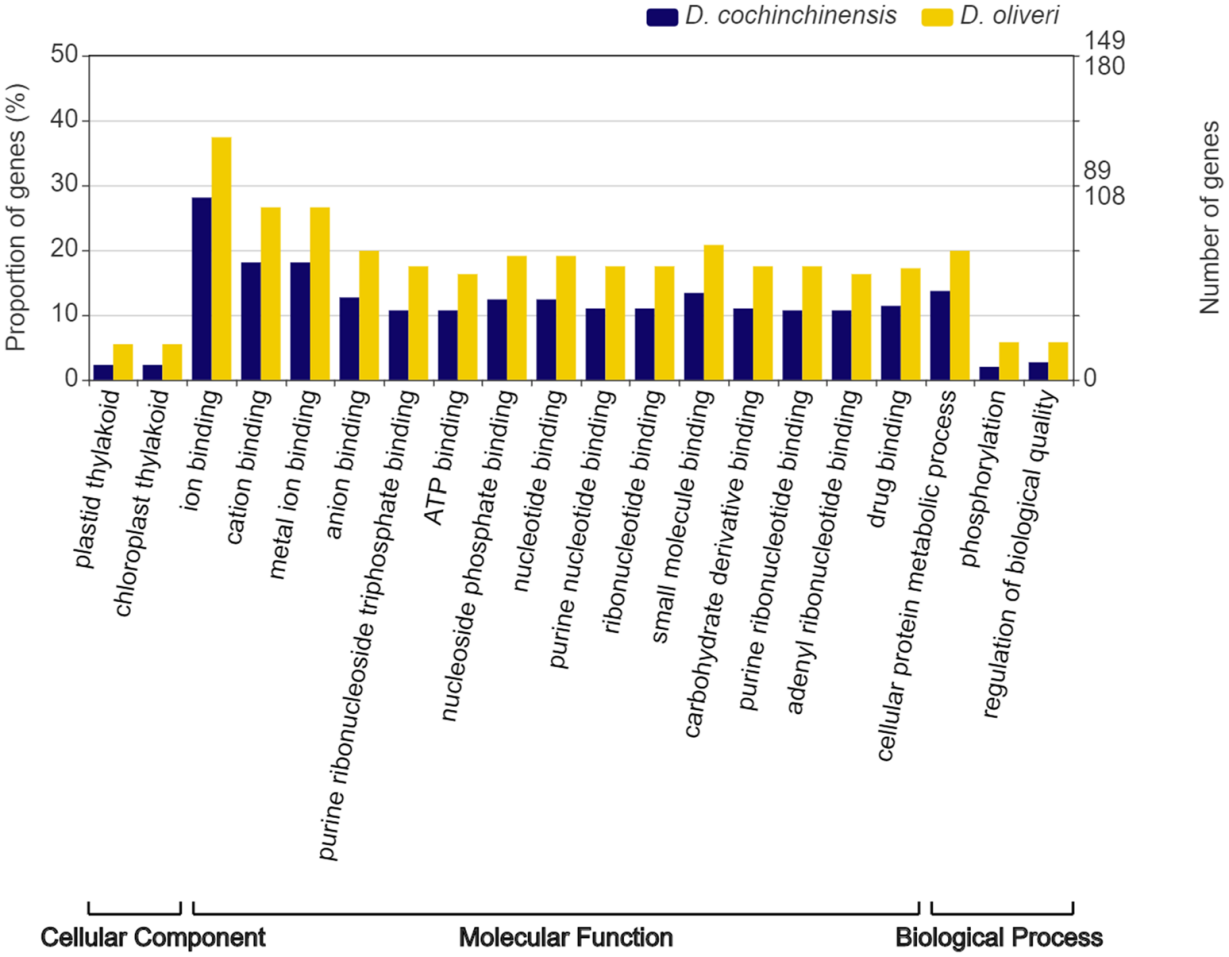
Results of GO enrichment analysis on positively selected genes, which are single-copy orthologs, between *D. cochinchinensis* (N = 371, GO annotated *n* = 299) and *D. oliveri* (N = 439, GO annotated *n* = 361), only showing terms that are significant (*p* < 0.05, chi-square test of independence).

A total of 9054 single-copy orthologs were identified among the 6 *Dalbergia* species using Orthofinder. A branch-site model, based on their *dN/dS*, detected 371 and 439 positively selected genes for *D. cochinchinensis* and *D. oliveri* respectively (BH *p* < 0.05, chi-square test of independence, Supplementary Table 11). KEGG and GO vocabularies were searched on these positively selected genes for individual species to better summarise their biological annotations. The GO enrichment test showed a significant difference between the two species in 20 level-6 GO terms (Fig. 4; *p* < 0.05, chi-square test of independence), with a majority of GO terms attributed to molecular function and related to binding. We detected no KEGG pathway or module showing a differential representation between these two species.

## Discussion

We produced 6 *Dalbergia* transcriptome assemblies estimated to each contain 32–49 K unique genes. Assessments of assembly completeness and quality suggested that they are suitable for molecular and evolutionary analyses and afford fair comparisons as presented in this study. Here, we discuss insights gained from data analyses with relevance to growth habit, divergence time and phylogeny, gene families, positive selection, and potential conservation implications.

### Transcriptome assembly statistics

Genome size variation has been an important character in the evolution of higher plants, and may be accompanied in some cases by substantial changes in the number of genes^61^. No genome has been published for the genus *Dalbergia*, but previous cytophotometry estimated that *Dalbergia* species have genome sizes ranging from 1.43–1.98 Gb, while *Dalbergia* is an exclusively diploid genus with 2*n* = 20 chromosomes^62^. Cytophotometry results also indicated a larger DNA content in climber or liana *Dalbergia* species than the congeneric tree species. A similar tree-liana evolution trend has been suggested in other woody angiosperm taxa^63,64^. A meta-study on 6949 angiosperms also confirmed that lianas generally have a larger DNA content^65^. In our study, *D. frustescens* was the only liana while others were all tree species. *D. frutescens* had the largest number of genes in its transcriptome, and was the most recently evolved, according to the most recent molecular phylogeny^1^. Both previous cytophotometry results and our transcriptome statistics suggest that the climbing character in *Dalbergia* may have derived from non-climbing tree ancestors, accompanied by both a larger genome size and an increased gene number. The expansion of gene families in lianas may underpin adaptations such as stem flexibility and vascular transport by adapted, derived secondary growth and wider vessel elements^66^. However, our study is limited by the number and choice of species, and we believe that studying more species in this large genus will give better insights into the tree-climber relationship.

### Phylogenomics and divergence time estimation

Molecular phylogenies have suggested *Dalbergia* is a monophyletic group placed in the *Dalbergia* clade with its sister taxon *Machaerium*^9,67^. The estimated age of MRCA of *Machaerium copote* and *Dalbergia congestiflora* was 40.4–43.0 MYA^52^. The most recent and comprehensive molecular phylogeny research in *Dalbergia* suggested *D. miscolobium* as the basal group among extant members^1^, but species divergence time in *Dalbergia* is unstudied to date. Using transcriptome resources and fossil calibrations from other Fabaceae species, we estimated the time of divergence of extant *Dalbergia* species to be around 14.78 MYA (Miocene-Langhian). Our estimation was slightly out of previously estimated ranges (^1^: 3.8–12.7 MYA and^68^: 7–12.2 MYA) based on single or a few loci. While most other fossil records of extinct members date to the Miocene (†*D. nostratum*: Lower Miocene 15.97–23.03 MYA^69^; †*D. lucida*: Late Miocene 5.33–11.61 MYA^70^), the earliest fossil record of †*D. phleboptera* was found in a Chattien (27.82–23.02 MYA) deposit^71^, which would suggest an earlier origin of the *Dalbergia* genus. However, the morphological details of extinct *Dalbergia* species were not well described from fossils and thus their placement within the genus *Dalbergia* could not be confirmed. Therefore, in our study, these *Dalbergia* fossils were not useful in node calibration to determine the actual divergence time of *Dalbergia*. We believe our *Dalbergia* crown age estimation would at least be useful in providing a minimum bound when phylogenomic information of other *Dalbergia* species becomes available.

The colonisation of *D. cochinchinensis* and *D. oliveri* in the Indochina biodiversity hotspot was estimated to occur ~ 11.68 MYA (Lower Miocene), coinciding with rapid *in-situ* diversification events and migrations after the Thai-Malay Peninsula split into Indochina and Borneo at ~ 15 MYA^72^, leading to Indochina’s diverse biota.

Divergence time for legumes was estimated to be ~ 80.16 MYA in this study, which falls within the most recent estimate of its marginal age prior (79.37–109.20 MYA)^73^. The difficulty in accurate divergence time estimation is proposed to be due to both whole genome duplication events near the root, intertwining with extinction and speciation events^73^.

### Comparative analysis of gene families between *Dalbergia* and other Fabaceae members

Eukaryotes share a large uniform set of conserved orthologs which encode for essential functional domains, such as DNA replication and repair, stress response, and secretion, and are based on the same genomic architeture^74^. The expansion and contraction of core orthologs contribute to eukaryotic diversity and enable individual species adaptation to their environment^75^. New genes may develop and result in the partitioning of gene function (subfunctionalisation) or the acquisition of new function (neofunctionalisation)^76^. For comparative genomic analyses of lineage-specific expansions and contractions, we used Pfam and CAFE analyses. The former tends to cluster protein into larger gene families, while the latter produces a finer clustering^59^.

Our Pfam analysis revealed expanded gene families in *Dalbergia* species compared to other Fabaceae members with potential biological relevance to their adaptive significance. For example, HSP70 and HSP90 heat shock proteins are molecular chaperones important for protein folding that enable active response to different stresses in plants such as heat, drought, pH and hypoxia via different signalling transduction pathways^77,78^. The protection against prolonged heat stress and acute heat shock by these chaperones has enabled heat acclimatization in *Arabidopsis thaliana*^79^, such as via stomatal control and abscisic acid signalling^80^. The expansion of HSPs in *Dalbergia* species may enhance their tolerance of higher temperatures across their pan-tropical range. Another significantly expanded protein family in the *Dalbergia* genus is the aldehyde dehydrogenase (ALDH) superfamily. ALDH is highly conserved in many metabolic pathways in higher plants and plays a significant role in aldehyde homoeostasis and redox balance^81^, such as in photorespiration and nitrate assimilation^82^. Increase in ALDH activity is shown to correlate with higher energy production, which fosters faster coleoptile elongation and seedling survival^83^. Many plant ALDH genes are also known to respond to a diversity of stresses including dehydration, heavy metals, salinity, and others^84^. Finally, several ubiquitin-related terms are over-represented in the *Dalbergia* genus. The best-characterised functions of ubiquitin proteins (Ub) are regulation of targeted protein degradation and maintenance of protein load in cells, with a role in manipulation of the proteome in response to abiotic stress conditions^85,86^. For example, an Ub was found to regulate the expression of heat shock proteins in *Brassica napus*^87^. In addition, Ubs can control pattern-recognition receptors, which are crucial for plant defence and immunity against pathogens^88^.

### Evolution of plant defence genes in *Dalbergia cochinchinensis* and *D. oliveri*

CAFE analysis was conducted to detect expanded gene families in *D. cochinchinensis* and *D. oliveri* compared to other *Dalbergia* and Fabaceae species. Both species showed a significant expansion in disease resistance proteins (R proteins): 34 R protein families were detected to expand in *D. oliveri* (294 R proteins), while 6 were detected in *D. cochinchinensis* (52 R proteins). GO enrichment of these significantly expanded gene families also confirmed an over-representation of immune response and defence response genes. R proteins are important in response to biotic stresses, as plants are attacked by many pathogenic organisms such as bacterial, fungi, viruses, and nematodes^89^. Pathogens secrete effector proteins during infection and can be recognised by R proteins in gene-for-gene interactions^90^. Due to the highly specific nature of R proteins on effectors, the R protein family evolves under diversifying selection for rapid acquisition of novel specificity to pathogens^91^.

Although *D. cochinchinensis* and *D. oliveri* are commonly found in the same geographical localities in Thailand, Laos, Cambodia and Vietnam, *D. oliveri* has a wider distribution towards Myanmar and occurs in a broader diversity of forest types^10^. The wider niche of *D. oliveri* may encompass a wider array of biotic stresses and diseases and thus explain the more diverged R protein families than in *D. cochinchinensis*.

Our PAML analysis detected 16 and 22 positively selected genes responsible for defence responses (GO: 0006952) in *D. cochinchinensis* and *D. oliveri,* respectively, suggesting an adaptive divergence in the suite of plant defence genes. Positive selection in PAML analysis is detected based on measuring the ratio of non-synonymous to synonymous substitution (*dN/dS*) for all single-copy orthologs, assuming *dN/dS* = 1 in neutral molecular evolution, *dN/dS* > 1 signals positive selection^92^. Most of the positively selected genes do not belong to the R family, but instead, for example, to the leucine-rich repeats (LRR) family, RNA-binding family, NPK1-related protein kinase family, which also are involved in the detection of pathogenic compounds and triggering of plant defence^93^.

Positive selection analysis also revealed several GO terms that were different between the two species, with *D. oliveri* having more positively selected genes in every term than *D. cochinchinesis*. Only 28 genes were positively selected in both *D. cochinchinensis* and *D. oliveri*, whereas they each had 343 and 411 positively selected distinct genes respectively. The difference in selection signals may suggest that even though the two species share similar geographical distributions, they are subject to different selective forces and slightly more genes have undergone positive selection in *D. oliveri* evolution. The only population genetic study revealed that *D. oliveri* maintains higher genetic diversity than *D. cochinchinensis* from ancient genetic bottlenecks, potentially related to higher gene flow and dispersal capacity in *D. oliveri*^10^. Potential selection differences between the two species will need further studies, such as through landscape genomics, to fully elucidate their gene-environment associations.

## Conclusion

Of the 14,191 vascular plants that have been listed as threatened (Vulnerable, Endangered and Critically Endangered) on the IUCN Red List (version February 2019)^94^, 16 (~ 0.1%) have published genomes and only 64 have published transcriptomes as BioProjects on NCBI (~ 0.5%)^95^. Compared to about 1% of threatened animal species with published genomes on NCBI^96^, there are disproportionately few genome-wide resources in threatened plants.

The potential application of genomic tools for conservation theory and practice has been clearly highlighted but its use is still limited in real-world initiatives^97^. One of the limitations is, assembling a reference genome involves considerable expertise, costs, and computational resources^98^. Advances in RNA-seq and transcriptomics offer a cost-effective alternative to facilitate diverse genomic applications^99^. Reference transcriptomes enable the development of an array of genotyping methods, such as microsatellites^96^, exon capture^100^, and SNP discovery with genotyping-by-sequencing^101^. Although targeted capture probes exist for legumes^102^, our transcriptomes capture a larger set of single or low-copy homologous genes exclusive to *Dalbergia*. The genome-wide resource allows us to study genetic diversity and understand both its neutral and adaptive components. This will produce insights into the mechanisms driving interactions between the environment and populations, with the potential to inform adaptive management of threatened populations, such as through assisted gene flow, GWAS, and marker-based or genomic selection^96,103^.

*Dalbergia* is highly threatened as a genus globally because of its economic value, with *D. cochinchinensis* and *D. oliveri* respectively characterised as Vulnerable and Engendered in the IUCN Red List. With overexploitation of these two species, timber markets have already shifted to other *Dalbergia* species leading to serial exploitation within the genus^104^*.* Our reference transcriptomes hugely expand the genomic resource repertoire for the genus *Dalbergia* and will facilitate transfer of utility through to other *Dalbergia* species. They will also open the potential for future studies of *Dalbergia* species towards their evolution and conservation in a broader context.

## Supplementary information

Supplementary Information.

Supplementary Data 1a.

Supplementary Data 1b.

Supplementary Data 1c.

Supplementary Data 1d.

Supplementary Data 1e.

Supplementary Data 1e.

Supplementary Table 8.

Supplementary Table 9a.

Supplementary Table 9b.

Supplementary Table 11a.

Supplementary Table 11b.

## Data Availability

The research materials supporting this publication can be publicly accessed either in the Supplementary Information or in NCBI GenBank under the BioProject PRJNA593817.
