# Supplementary material for: Reference transcriptomes and comparative analyses of six species in the threatened rosewood genus Dalbergia
Source: Sci Rep. 2020 Oct 20;10:17749. doi: 10.1038/s41598-020-74814-2 (PMC7576600; doi:10.1038/s41598-020-74814-2)
Supplement: Supplementary file 1 — Supplementary Information. [file 41598_2020_74814_MOESM1_ESM.docx]

*Supplementary Materials*

Reference transcriptomes and comparative analyses of six species in the threatened rosewood genus *Dalbergia*

Tin Hang Hung^1,*^, Thea So^2^, Syneath Sreng^2^, Bansa Thammavong^3^, Chaloun Boounithiphonh^3^, David H. Boshier^1^, John J. MacKay^1,*^

1. Department of Plant Sciences, University of Oxford, Oxford OX1 3RB, United Kingdom

2. Institute of Forest and Wildlife Research and Development, Cambodia

3. Forest Research Center, National Agriculture and Forestry Research Institute, Lao PDR

Corresponding authors:

*T.H.H.: [tin-hang.hung@plants.ox.ac.uk](mailto:tin-hang.hung@plants.ox.ac.uk)

*J.J.M.: [john.mackay@plants.ox.ac.uk](mailto:john.mackay@plants.ox.ac.uk)

**Keywords:** transcriptomes, *Dalbergia*, rosewoods, RNA-seq

**Running title:** Reference transcriptomes of *6 Dalbergia* species

**Supplementary Table 1.** Acquisition details for the plant materials of the 6 *Dalbergia* species covered in this study**.**

**Supplementary Table 2.** Transcriptome and annotation resources of the 16 Fabaceae species used in this study.

**Supplementary Table 3.** Summary of transcriptome BUSCO statistics (N = 2,121) of the 6 *Dalbergia* species

**Supplementary Figure 1.** Transcripts of the 6 *Dalbergia* species aligned to the *Arachis ipaensis* NCBI RefSeq database at a given coverage.

**Supplementary Dataset 1.** Trinotate annotation reports of (a) *D. cochinchinensis*, (b) *D. frutescens*, (c) *D. melanoxylon*, (d) *D. miscolobium*, (e) *D. oliveri*, (f) *D. sissoo*. For the versions of annotation databases, see methods for details.

**Supplementary Table 4.** TransRate reference-based metrics of the 6 *Dalbergia* transcriptomes mapped on *Arachis ipaensis*. CRBB means Conditional Reciprocal Best BLAST. RBH means reciprocal best hit.

**Supplementary Table 5.** (a) Basic statistics of the Orthofinder results of the 16 Fabaceae species and (b) the number of shared orthogroups among species. See Supplementary Table 2 for species abbreviations.

**Supplementary Figure 2.** Venn diagram showing orthogroups among the 6 *Dalbergia* species visualized with ClusterVenn.

**Supplementary Table 6.** Estimation of node ages in the phylogeny of 16 Fabaceae species based on Bayesian analysis of a supergene from the 256 single-copy orthologs (479,064 bp) from their transcriptomes.

**Supplementary Table 7.** Counts of annotated GO terms (level 2) in 6 *Dalbergia* species, only showing terms that are significant different among the species (p-value < 0.05 in chi-square test of independence). See Supplementary Table 2 for species abbreviations.

**Supplementary Figure 3.** Chord diagrams of annotated GO terms (level 2) for (a) cellular components (red); (b) biological processes (green); (c) molecular functions (blue) in the 6 *Dalbergia* species, only showing terms that are significantly different among the species (p < 0.05 in chi-square test of independence). Each tick interval is 1,000 counts. The scale is arranged clockwise showing descending GO counts. See Supplementary Table 2 for species abbreviations.

**Supplementary Table 8.** Row Z scores of annotated Pfam domains (N = 17,929) of the 13 Fabaceae species, only showing domains (n = 91) that are significantly contracted (negative) or expanded (positive) in the *Dalbergia* species (p < 0.05 in two-tailed Fisher’s exact test of independence). See Supplementary Table 2 for species abbreviations.

**Supplementary Table 9.** Results of CAFE analysis on gene expansion/contraction events in (a) *D. cochinchinensis* and (b) *D. oliveri*, only showing 10 and 49 orthogroups that have significantly expanded or contracted (family p < 0.05 and Viterbi p < 0.001).

**Supplementary Table 10.** GO enrichment for biological processes of significantly expanded gene families in CAFE analysis in (a) *D. cochinchinensis* and (b) *D. oliveri*, only showing GO terms that are significantly over- or under-presented (p < 0.05 in two-tailed Fisher’s exact test of independence).

**Supplementary Table 11.** Results of PAML analysis on positive selection in (a) *D. cochinchinensis* and (b) *D. oliveri*, detecting 371 and 439 positively selected genes respectively, out of 9,054 single-copy orthologues tested (BH p < 0.05 in chi-square test of independence).

Supplementary Table 1

| **Species** | **Source** | **Accession** | **Year collected** | **Wild** | **Identifier used by source** |
| --- | --- | --- | --- | --- | --- |
| *D. cochinchinensis* | Forest Research Center, Lao PDR | Bolikhamxay, Khamkend, Laos | 2018 | Yes | N/A |
| *D. frutescens* | Millennium Seed Bank, Royal Botanic Gardens, Kew | Brazil | 1992 | Yes | 98382 |
| *D. melanoxylon* | World Agroforestry Centre | Kathozweni, Kenya | 2011 | Yes | 05662 |
| *D. miscolobium* | Royal Botanic Gardens, Kew | Brazil | 1994 | Yes | 102498 |
| *D. oliveri* | Institute of Forest & Wildlife Research & Development, Cambodia | Cambodia | 2018 | Yes | N/A |
| *D. sissoo* | Commercial retailer (http://www.rarepalmseeds.com) | Unknown | Unknown | Unknown | YRFFDASI |

Supplementary Table 2

| **Species** | **Abbreviation used in this paper** | **Source** | **Assembly method** | **BUSCO score** | **Reference** |
| --- | --- | --- | --- | --- | --- |
| ***Acrocarpus fraxinifolius*** | Af | https://ics.hutton.ac.uk/tropiTree/ | Trinity | Unreported | ^1^ |
| ***Arachis ipaensis*** | Ai | NCBI: GCF_000816755.2 | Trinity | C: 87.3; F: 5.8; M: 6.9 | ^2^ |
| ***Arachis duranensis*** | Ad | NCBI: GCF_000817695.2 | Trinity | C: 88.8; F: 4.7; M: 6.5 | ^2^ |
| ***Bauhinia tomentosa*** | Bt | DOI: 10.5061/dryad.ff1tq | Trinity | Unreported | ^3^ |
| ***Cercis canadensis*** | Cc | DOI: 10.5524/101044 | Trinity | C: 96.2; F: 1.9; M: 1.9 | ^4^ |
| ***Chamaecrista fasciculata*** | Cf | DOI: 10.5524/101045 | Trinity | C: 93.2; F: 1.7; M: 5.1 | ^4^ |
| ***Dalbergia cochinchinensis*** | Co | NCBI: GIHU00000000 | Trinity | C: 92.2; F: 4.0; M: 2.9 | this study |
| ***Dalbergia frutescens*** | Fr | NCBI: GIHP00000000 | Trinity | C: 92.1; F: 4.8; M: 3.1 | this study |
| ***Dalbergia melanoxylon*** | Me | NCBI: GIHQ00000000 | Trinity | C: 92.3; F: 5.1; M: 2.6 | this study |
| ***Dalbergia miscolobium*** | Mi | NCBI: GIHR00000000 | Trinity | C: 93.1; F: 4.6; M: 2.3 | this study |
| ***Dalbergia oliveri*** | Ol | NCBI: GIHS00000000 | Trinity | C: 90.9; F. 6.5; M: 2.6 | this study |
| ***Dalbergia sissoo*** | Si | NCBI: GIHT00000000 | Trinity | C; 94.4; F: 3.3; M: 2.3 | this study |
| ***Mimosa pudica*** | Mp | DOI: 10.5524/101049 | Trinity | C: 94.3; F: 1.7; M: 4.0 | ^4^ |
| ***Lupinus angustifolius*** | La | NCBI: GCF_001865875.1 | Trinity | C: 92.7; F: 4.2; M: 3.1 | ^5^ |
| ***Nissolia schottii*** | Ns | DOI:10.5524/101050 | Trinity | C: 95.0; F: 1.5; M: 3.5 | ^4^ |
| ***Xanthocercis zambesiaca*** | Xz | DOI: 10.5061/dryad.ff1tq | Trinity | Unreported | ^3^ |

Supplementary Table 3

| **BUSCO statistics** | ***D. cochinchinensis*** | ***D. frutescens*** | ***D. melanoxylon*** | ***D. miscolobium*** | ***D. oliveri*** | ***D. sissoo*** |
| --- | --- | --- | --- | --- | --- | --- |
| **Complete** | 1,956 (92.2%) | 1,953 (92.1%) | 1,958 (92.3%) | 1,976 (93.1%) | 1,927 (90.9%) | 2,002 (94.4%) |
| ***single-copy*** | 1,180 (55.6%) | 1,257 (59.3%) | 1,236 (58.3%) | 1,254 (59.1%) | 1,172 (55.3%) | 1,331 (62.8%) |
| ***duplicated*** | 776 (36.6%) | 696 (32.8%) | 722 (34.0%) | 722 (34.0%) | 755 (35.6%) | 671 (31.6%) |
| **Fragmented** | 104 (4.9%) | 102 (4.8%) | 109 (5.1%) | 97 (4.6%) | 137 (6.5%) | 69 (3.3%) |
| **Missing** | 61 (2.9%) | 66 (3.1%) | 54 (2.6%) | 48 (2.3%) | 57 (2.6%) | 50 (2.3%) |

Supplementary Figure 1


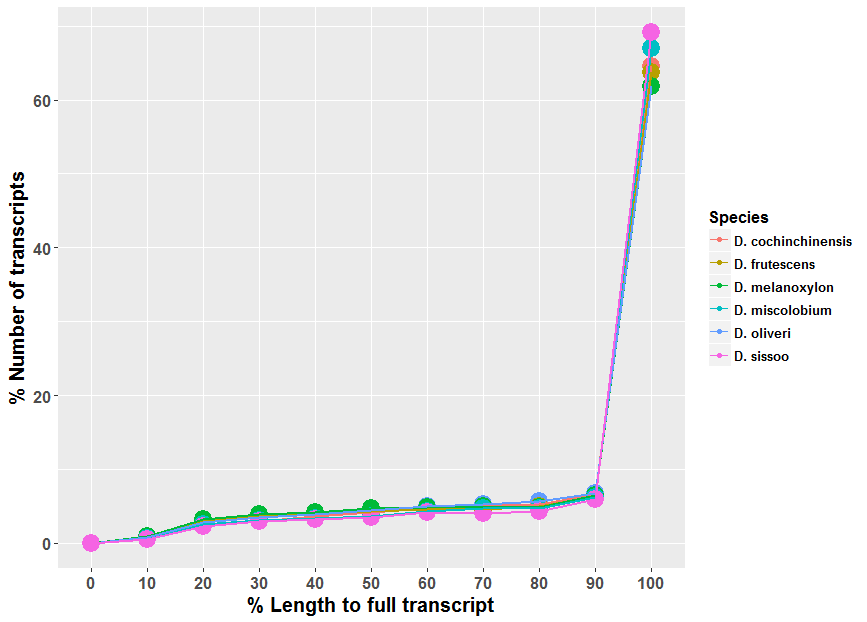
 Supplementary Dataset 1

(a) In a separate spreadsheet

SD1a - Dalbergia cochinchinensis annotation report.xlsx

(a) In a separate spreadsheet

SD1b - Dalbergia frutescens annotation report.xlsx

(a) In a separate spreadsheet

SD1c - Dalbergia melanoxylon annotation report.xlsx

(a) In a separate spreadsheet

SD1d - Dalbergia miscolobium annotation report.xlsx

(a) In a separate spreadsheet

SD1e - Dalbergia oliveri annotation report.xlsx

(a) In a separate spreadsheet

SD1f - Dalbergia sissoo annotation report.xlsx

Supplementary Table 4

| **Feature** | ***D. cochinchinensis*** | ***D. frutescens*** | ***D. melanoxylon*** | ***D. miscolobium*** | ***D. oliveri*** | ***D. sissoo*** |
| --- | --- | --- | --- | --- | --- | --- |
| **Number of sequences** | 34,655 | 48,591 | 43,848 | 31,678 | 43,879 | 32,753 |
| **Number of contigs with an ORF** | 23,440 | 30,717 | 26,763 | 22,084 | 26,737 | 22,887 |
| **Mean % of the contig covered by ORF** | 99.78 | 99.80 | 99.79 | 99.79 | 99.79 | 99.78 |
| **CRBB hit** | 14,963 | 14,820 | 15,668 | 14,778 | 15,395 | 14,365 |
| **Number of contigs with CRBB** | 14,963 (43.18%) | 14,820 (30.50%) | 15,668 (35.73%) | 14,778 (46.65%) | 15,395 (35.09%) | 14,365 (43.86%) |
| **RBH per reference (%)** | 35.76 | 35.42 | 37.45 | 35.32 | 36.80 | 34.33 |
| **Number of references with CRBB** | 13,465 (32.18%) | 13,405 (32.04%) | 13,649 (32.62%) | 13,419 (32.07%) | 13,618 (32.55%) | 13,196 (31.54%) |
| **Reference coverage (%)** | 34.38 | 34.21 | 34.47 | 34.43 | 34.42 | 34.10 |

Supplementary Table 5

**(a)**

| **Number of genes** | 568,554 |
| --- | --- |
| **Number of genes in orthogroups** | 481,614 (84.7%) |
| **Number of orthogroups** | 34,725 |
| **Number of species-specific orthogroups** | 925 |
| **Number of genes in species-specific orthogroups** | 3,465 (0.6%) |
| **Number of orthogroups with all species present** | 5,493 |
| **Number of single-copy orthogroups** | 256 |

**(b)**

|  | Ad | Af | Ai | Bt | Cc | Cf | Co | Fr | Me | Mi | Ol | Si | La | Mp | Ns | Xz |
| --- | --- | --- | --- | --- | --- | --- | --- | --- | --- | --- | --- | --- | --- | --- | --- | --- |
| Ad | 16,595 | 10,149 | 15,648 | 10,595 | 12,256 | 11,498 | 12,346 | 12,337 | 12,501 | 12,221 | 12,441 | 12,266 | 12,013 | 10,540 | 12,416 | 10,806 |
| Af | 10,149 | 13,646 | 10,253 | 10,781 | 10,374 | 9,791 | 10,946 | 11,281 | 11,440 | 10,991 | 11,232 | 11,005 | 10,253 | 9,101 | 10,245 | 10,970 |
| Ai | 15,648 | 10,253 | 16,834 | 10,687 | 12,283 | 11,528 | 12,429 | 12,431 | 12,614 | 12,311 | 12,529 | 12,319 | 12,034 | 10,595 | 12,431 | 10,892 |
| Bt | 10,595 | 10,781 | 10,687 | 13,812 | 11,051 | 10,261 | 11,461 | 11,598 | 11,843 | 11,445 | 11,668 | 11,371 | 10,750 | 9,520 | 10,811 | 11,423 |
| Cc | 12,256 | 10,374 | 12,283 | 11,051 | 14,960 | 12,393 | 12,848 | 12,832 | 12,973 | 12,711 | 12,938 | 12,710 | 12,589 | 11,261 | 13,112 | 11,095 |
| Cf | 11,498 | 9,791 | 11,528 | 10,261 | 12,393 | 13,822 | 12,032 | 12,009 | 12,138 | 11,931 | 12,110 | 11,922 | 11,892 | 10,813 | 12,301 | 10,372 |
| Co | 12,346 | 10,946 | 12,429 | 11,461 | 12,848 | 12,032 | 18,209 | 15,388 | 15,739 | 14,696 | 16,060 | 14,769 | 12,475 | 11,052 | 13,022 | 11,754 |
| Fr | 12,337 | 11,281 | 12,431 | 11,598 | 12,832 | 12,009 | 15,388 | 21,650 | 17,347 | 15,693 | 17,047 | 16,873 | 12,464 | 11,003 | 12,978 | 11,925 |
| Me | 12,501 | 11,440 | 12,614 | 11,843 | 12,973 | 12,138 | 15,739 | 17,347 | 20,719 | 15,388 | 16,678 | 16,007 | 12,568 | 11,128 | 13,128 | 12,224 |
| Mi | 12,221 | 10,991 | 12,311 | 11,445 | 12,711 | 11,931 | 14,696 | 15,693 | 15,388 | 17,963 | 15,467 | 14,862 | 12,397 | 10,925 | 12,875 | 11,762 |
| Ol | 12,441 | 11,232 | 12,529 | 11,668 | 12,938 | 12,110 | 16,060 | 17,047 | 16,678 | 15,467 | 20,592 | 15,650 | 12,547 | 11,132 | 13,158 | 11,949 |
| Si | 12,266 | 11,005 | 12,319 | 11,371 | 12,710 | 11,922 | 14,769 | 16,873 | 16,007 | 14,862 | 15,650 | 19,090 | 12,394 | 10,939 | 12,882 | 11,681 |
| La | 12,013 | 10,253 | 12,034 | 10,750 | 12,589 | 11,892 | 12,475 | 12,464 | 12,568 | 12,397 | 12,547 | 12,394 | 13,882 | 10,826 | 12,668 | 10,902 |
| Mp | 10,540 | 9,101 | 10,595 | 9,520 | 11,261 | 10,813 | 11,052 | 11,003 | 11,128 | 10,925 | 11,132 | 10,939 | 10,826 | 12,882 | 11,254 | 9,592 |
| Ns | 12,416 | 10,245 | 12,431 | 10,811 | 13,112 | 12,301 | 13,022 | 12,978 | 13,128 | 12,875 | 13,158 | 12,882 | 12,668 | 11,254 | 15,005 | 11,024 |
| Xz | 10,806 | 10,970 | 10,892 | 11,423 | 11,095 | 10,372 | 11,754 | 11,925 | 12,224 | 11,762 | 11,949 | 11,681 | 10,902 | 9,592 | 11,024 | 14,319 |

Supplementary Figure 2

**
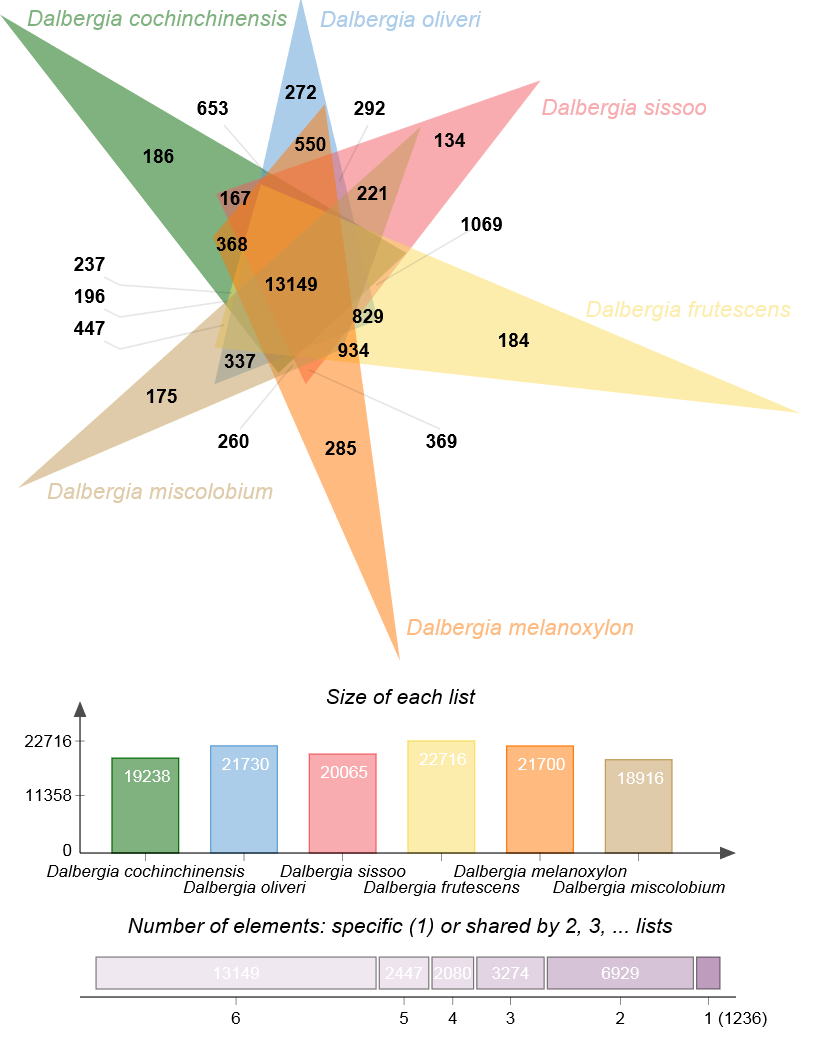
**

Supplementary Table 6

| **Node** | **Median of node age** | **95% HPD of node age** |
| --- | --- | --- |
| *Bauhinia tomentosa–Acrocarpus fraxinifolius* | 80.1610 | 69.4302 – 95.4551 |
| *Bauhinia tomentosa–Cercis canadensis* | 46.8064 | 43.4005 – 50.6522 |
| *Acrocarpus fraxinifolius–Chamaecrista fasciculata* | 58.2181 | 56.9537 – 59.8703 |
| *Chamaecrista fasciculata–Mimosa pudica* | 43.8327 | 42.2482 – 46.0581 |
| *Acrocarpus fraxinifolius–Xanthocercis zambesiaca* | 64.0946 | 62.6783 – 65.9590 |
| *Xanthocercis zambesiaca–Lupinus augustifolius* | 57.1341 | 56.2045 – 58.7156 |
| *Lupinus augustifolius–Nissolia schottii* | 51.3041 | 50.4486 – 52.5991 |
| *Nissolia schottii–Arachis duranensis* | 41.8895 | 41.1156 – 42.8862 |
| *Arachis duranensis–Arachis ipaensis* | 3.2624 | 3.1225 – 3.4102 |
| *Arachis duranensis–Dalbergia miscolobium* | 25.5414 | 24.0776 – 27.1138 |
| *Dalbergia miscolobium–Dalbergia cochinchinensis* | 14.7808 | 13.7365 – 16.0182 |
| *Dalbergia cochinchinensis–Dalbergia oliveri* | 11.6900 | 9.6624 – 13.6183 |
| *Dalbergia cochinchinensis–Dalbergia frutescens* | 12.7026 | 10.4926 – 14.4774 |
| *Dalbergia frutescens–Dalbergia sissoo* | 10.0446 | 7.7557 – 12.8381 |
| *Dalbergia frutescens–Dalbergia melanoxylon* | 7.0789 | 4.8187 – 10.2959 |

Supplementary Table 7

|  | **Co** | **Fr** | **Me** | **Mi** | **Ol** | **Si** | **p-value** |
| --- | --- | --- | --- | --- | --- | --- | --- |
| ***Cellular components*** |  |  |  |  |  |  |  |
| cell | 18918 | 25665 | 22120 | 18346 | 22471 | 18756 | 0.00E+00 |
| cell junction | 1056 | 1071 | 1166 | 986 | 1266 | 934 | 3.53E-13 |
| cell part | 18887 | 25610 | 22084 | 18305 | 22427 | 18715 | 0.00E+00 |
| extracellular region | 1587 | 2179 | 1650 | 1567 | 1819 | 1544 | 2.20E-35 |
| extracellular region part | 370 | 392 | 308 | 345 | 415 | 302 | 2.30E-05 |
| membrane | 8770 | 11238 | 10013 | 8320 | 10102 | 8345 | 2.88E-155 |
| membrane-enclosed lumen | 1495 | 2825 | 1876 | 1544 | 1859 | 1752 | 2.01E-131 |
| membrane part | 6488 | 8413 | 7749 | 6105 | 7521 | 6151 | 8.28E-138 |
| nucleoid | 47 | 91 | 76 | 66 | 61 | 79 | 4.67E-03 |
| organelle | 13715 | 18843 | 15907 | 13489 | 16265 | 13898 | 5.45E-302 |
| organelle part | 6824 | 10183 | 8093 | 6886 | 8407 | 7025 | 3.76E-229 |
| protein-containing complex | 3441 | 5836 | 4096 | 3677 | 4551 | 3818 | 1.52E-191 |
| supramolecular complex | 363 | 416 | 344 | 369 | 437 | 343 | 1.20E-03 |
| symplast | 991 | 981 | 1120 | 936 | 1166 | 878 | 1.34E-11 |
| synapse | 82 | 121 | 79 | 85 | 130 | 68 | 3.05E-06 |
| synapse part | 67 | 96 | 65 | 68 | 103 | 57 | 3.22E-04 |
| ***Molecular functions*** |  |  |  |  |  |  |  |
| antioxidant activity | 199 | 290 | 218 | 201 | 247 | 234 | 1.27E-04 |
| binding | 16104 | 21436 | 19157 | 15169 | 19352 | 15228 | 0.00E+00 |
| cargo receptor activity | 3 | 19 | 11 | 6 | 10 | 5 | 2.44E-03 |
| catalytic activity | 11947 | 16786 | 14322 | 11596 | 14272 | 12024 | 0.00E+00 |
| molecular carrier activity | 38 | 85 | 57 | 42 | 48 | 55 | 9.03E-05 |
| molecular function regulator | 526 | 763 | 566 | 519 | 634 | 421 | 4.79E-24 |
| molecular transducer activity | 471 | 527 | 547 | 438 | 512 | 562 | 5.90E-04 |
| structural molecule activity | 843 | 1233 | 836 | 908 | 1301 | 778 | 1.90E-53 |
| transcription regulator activity | 1552 | 1839 | 1745 | 1487 | 1683 | 1512 | 7.30E-12 |
| transporter activity | 1608 | 2477 | 1954 | 1651 | 1863 | 1710 | 1.71E-57 |
| ***Biological processes*** |  |  |  |  |  |  |  |
| behavior | 26 | 45 | 25 | 31 | 46 | 16 | 4.40E-04 |
| biological adhesion | 76 | 121 | 84 | 78 | 100 | 66 | 4.07E-04 |
| biological regulation | 6865 | 8847 | 7968 | 6534 | 8031 | 6527 | 1.11E-130 |
| cell aggregation | 34 | 31 | 10 | 46 | 52 | 21 | 5.27E-07 |
| cellular component organization or biogenesis | 4070 | 6294 | 4680 | 4021 | 4912 | 4280 | 6.98E-164 |
| cellular process | 15997 | 22257 | 18818 | 15510 | 19098 | 15839 | 0.00E+00 |
| detoxification | 58 | 149 | 100 | 83 | 82 | 85 | 1.06E-09 |
| developmental process | 3630 | 4014 | 4035 | 3567 | 4074 | 3411 | 9.97E-22 |
| growth | 778 | 925 | 881 | 730 | 878 | 721 | 8.57E-09 |
| immune system process | 1108 | 1217 | 1379 | 849 | 1394 | 760 | 1.88E-66 |
| localization | 3349 | 5211 | 3926 | 3409 | 4058 | 3535 | 2.10E-131 |
| locomotion | 173 | 233 | 156 | 217 | 254 | 169 | 1.86E-07 |
| metabolic process | 13803 | 19567 | 16249 | 13673 | 16395 | 14048 | 0.00E+00 |
| multi-organism process | 2627 | 2901 | 2935 | 2303 | 3053 | 2180 | 2.15E-50 |
| multicellular organismal process | 3462 | 3571 | 3866 | 3352 | 3822 | 3138 | 3.68E-22 |
| negative regulation of biological process | 1462 | 2104 | 1747 | 1478 | 1715 | 1519 | 8.36E-37 |
| nitrogen utilization | 15 | 58 | 25 | 16 | 24 | 14 | 1.36E-10 |
| positive regulation of biological process | 1745 | 2403 | 1977 | 1713 | 2057 | 1756 | 2.33E-37 |
| regulation of biological process | 6179 | 7853 | 7137 | 5833 | 7183 | 5816 | 6.84E-113 |
| reproduction | 2037 | 2244 | 2276 | 1955 | 2234 | 1849 | 8.84E-15 |
| reproductive process | 2014 | 2191 | 2254 | 1929 | 2201 | 1811 | 7.93E-15 |
| response to stimulus | 7706 | 9228 | 8974 | 6972 | 8989 | 6820 | 1.18E-154 |
| signaling | 2553 | 3053 | 2987 | 2219 | 3069 | 2086 | 3.99E-76 |

Supplementary Figure 3

(a)


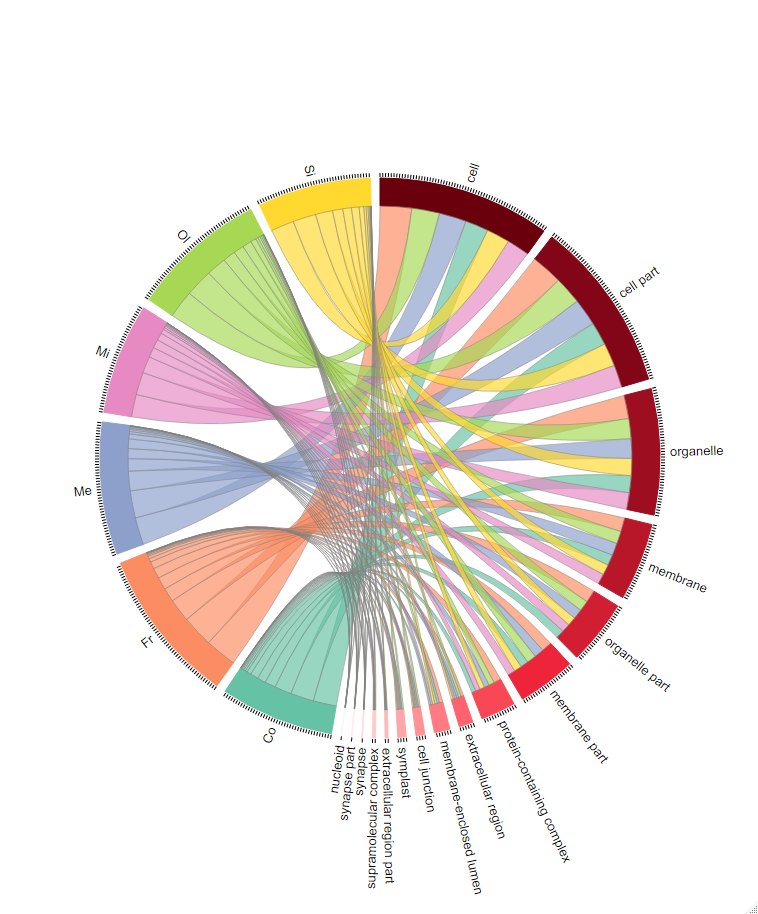


(b)


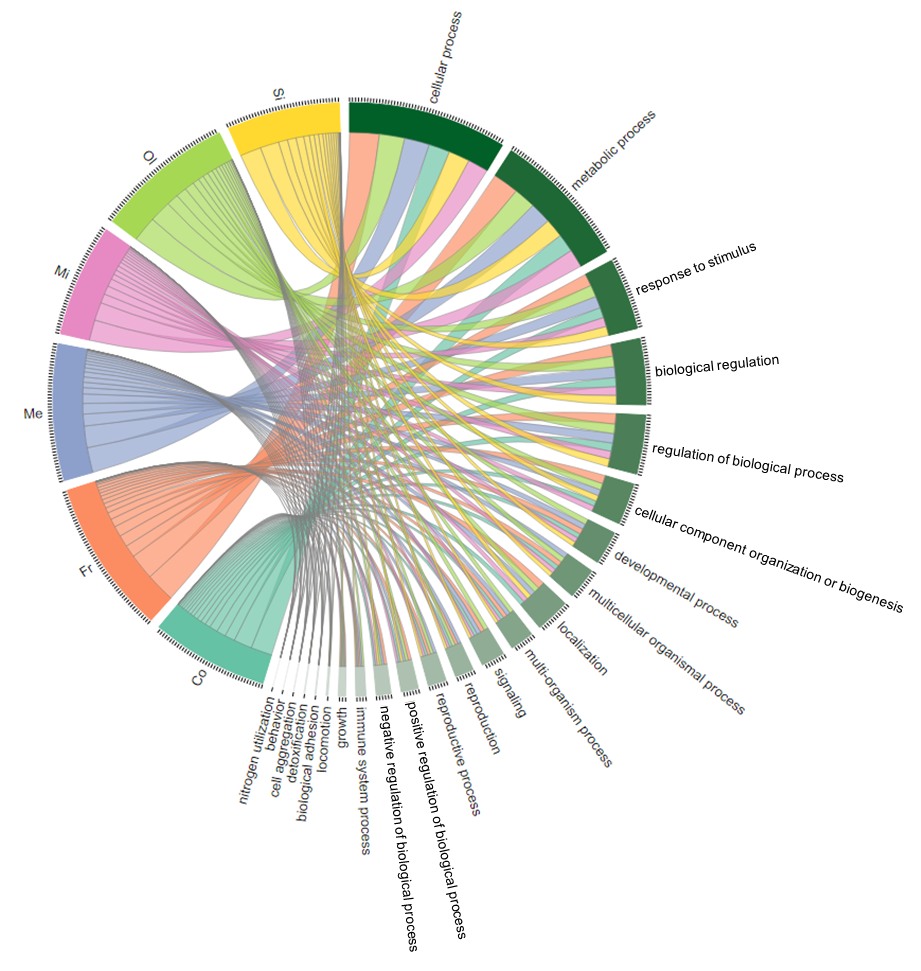


(c)


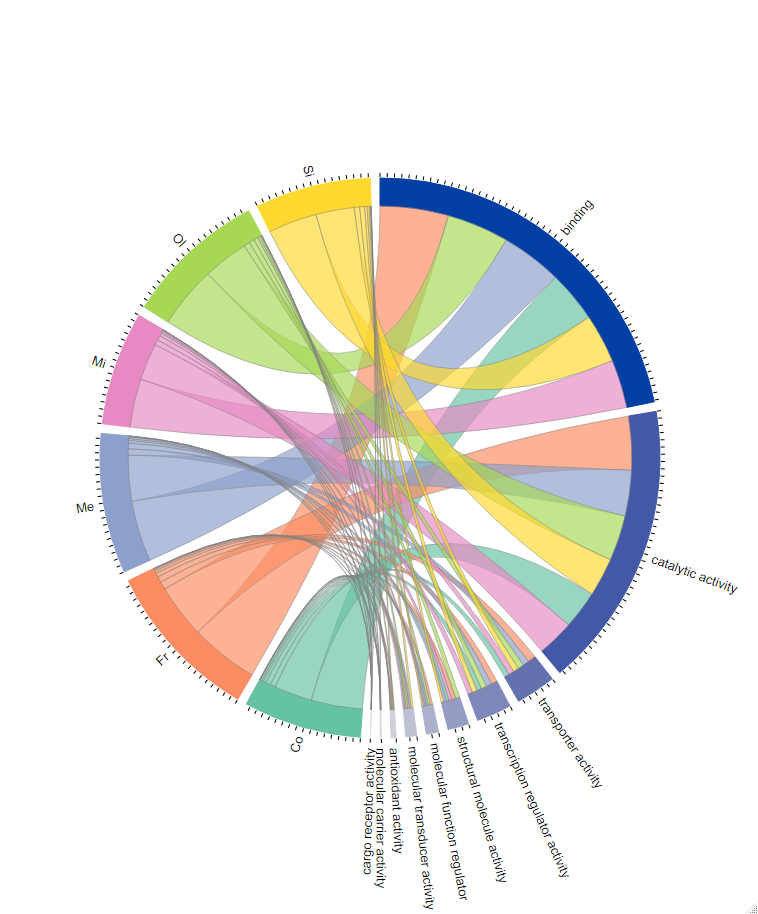


Supplementary Table 8

(a) In a separate spreadsheet

ST8 - Row Z scores of Pfam terms.xlsx

Supplementary Table 9

(a) In a separate spreadsheet

ST9a - CAFE analysis of D. cochinchinensis.xlsx

(b) In a separate spreadsheet

ST9b - CAFE analysis of D. oliveri.xlsx

Supplementary Table 10a

| **GO biological process** | ***Arabidopsis thaliana* (27416)** | ***D. cochinchinensis* (71)** | **Expected** | **Over/under** | **Fold enrichment** | **p-value** |
| --- | --- | --- | --- | --- | --- | --- |
| recognition of pollen (GO:0048544) | 48 | 5 | 0.12 | + | 40.22 | 7.41E-04 |
| cell recognition (GO:0008037) | 50 | 5 | 0.13 | + | 38.61 | 8.94E-04 |
| pollen-pistil interaction (GO:0009875) | 62 | 5 | 0.16 | + | 31.14 | 2.42E-03 |
| immune system process (GO:0002376) | 361 | 9 | 0.93 | + | 9.63 | 1.34E-03 |
| innate immune response (GO:0045087) | 306 | 7 | 0.79 | + | 8.83 | 4.72E-02 |
| defense response to bacterium (GO:0042742) | 413 | 9 | 1.07 | + | 8.41 | 3.99E-03 |
| signal transduction (GO:0007165) | 1330 | 27 | 3.44 | + | 7.84 | 4.22E-14 |
| cell communication (GO:0007154) | 1618 | 32 | 4.19 | + | 7.64 | 2.77E-17 |
| response to bacterium (GO:0009617) | 506 | 10 | 1.31 | + | 7.63 | 2.35E-03 |
| signaling (GO:0023052) | 1369 | 27 | 3.55 | + | 7.62 | 8.58E-14 |
| protein phosphorylation (GO:0006468) | 1037 | 20 | 2.69 | + | 7.45 | 4.18E-09 |
| defense response to other organism (GO:0098542) | 805 | 15 | 2.08 | + | 7.2 | 6.41E-06 |
| phosphorylation (GO:0016310) | 1216 | 20 | 3.15 | + | 6.35 | 7.07E-08 |
| defense response (GO:0006952) | 952 | 15 | 2.47 | + | 6.08 | 5.81E-05 |
| response to external biotic stimulus (GO:0043207) | 1092 | 15 | 2.83 | + | 5.3 | 3.40E-04 |
| response to other organism (GO:0051707) | 1092 | 15 | 2.83 | + | 5.3 | 3.40E-04 |
| response to biotic stimulus (GO:0009607) | 1093 | 15 | 2.83 | + | 5.3 | 3.44E-04 |
| interspecies interaction between organisms (GO:0044419) | 1120 | 15 | 2.9 | + | 5.17 | 4.70E-04 |
| phosphate-containing compound metabolic process (GO:0006796) | 1845 | 20 | 4.78 | + | 4.19 | 8.68E-05 |
| cellular response to stimulus (GO:0051716) | 2521 | 27 | 6.53 | + | 4.14 | 1.63E-07 |
| response to external stimulus (GO:0009605) | 1508 | 16 | 3.91 | + | 4.1 | 3.71E-03 |
| phosphorus metabolic process (GO:0006793) | 1890 | 20 | 4.89 | + | 4.09 | 1.29E-04 |
| cellular protein metabolic process (GO:0044267) | 3370 | 28 | 8.73 | + | 3.21 | 2.03E-05 |
| protein modification process (GO:0036211) | 2496 | 20 | 6.46 | + | 3.09 | 1.04E-02 |
| cellular protein modification process (GO:0006464) | 2496 | 20 | 6.46 | + | 3.09 | 1.04E-02 |
| protein metabolic process (GO:0019538) | 3539 | 28 | 9.17 | + | 3.06 | 5.96E-05 |
| macromolecule modification (GO:0043412) | 2813 | 22 | 7.28 | + | 3.02 | 4.27E-03 |
| cellular macromolecule metabolic process (GO:0044260) | 4404 | 30 | 11.41 | + | 2.63 | 4.36E-04 |
| response to stimulus (GO:0050896) | 5567 | 36 | 14.42 | + | 2.5 | 3.68E-05 |
| regulation of cellular process (GO:0050794) | 4726 | 29 | 12.24 | + | 2.37 | 1.11E-02 |
| organonitrogen compound metabolic process (GO:1901564) | 4613 | 28 | 11.95 | + | 2.34 | 2.05E-02 |
| regulation of biological process (GO:0050789) | 5374 | 30 | 13.92 | + | 2.16 | 4.07E-02 |
| cellular process (GO:0009987) | 11959 | 55 | 30.97 | + | 1.78 | 2.81E-05 |
| Unclassified (UNCLASSIFIED) | 5470 | 10 | 14.17 | - | 0.71 | 0.00E+00 |

Supplementary Table 10b

| **GO biological process** | ***Arabidopsis thaliana* (27416)** | ***D. oliveri* (254)** | **Expected** | **Over/under** | **Fold enrichment** | **p-value** |
| --- | --- | --- | --- | --- | --- | --- |
| autophagosome maturation (GO:0097352) | 3 | 3 | 0.03 | + | > 100 | 4.27E-02 |
| spindle disassembly (GO:0051230) | 3 | 3 | 0.03 | + | > 100 | 4.27E-02 |
| mitotic spindle disassembly (GO:0051228) | 3 | 3 | 0.03 | + | > 100 | 4.27E-02 |
| protein refolding (GO:0042026) | 32 | 9 | 0.3 | + | 30.36 | 3.11E-07 |
| immune response-regulating signaling pathway (GO:0002764) | 20 | 5 | 0.19 | + | 26.98 | 8.17E-03 |
| cellular response to unfolded protein (GO:0034620) | 41 | 9 | 0.38 | + | 23.69 | 2.07E-06 |
| response to unfolded protein (GO:0006986) | 46 | 9 | 0.43 | + | 21.12 | 5.04E-06 |
| chaperone cofactor-dependent protein refolding (GO:0051085) | 47 | 9 | 0.44 | + | 20.67 | 5.96E-06 |
| 'de novo' posttranslational protein folding (GO:0051084) | 47 | 9 | 0.44 | + | 20.67 | 5.96E-06 |
| cellular response to topologically incorrect protein (GO:0035967) | 65 | 12 | 0.6 | + | 19.93 | 1.71E-08 |
| 'de novo' protein folding (GO:0006458) | 49 | 9 | 0.45 | + | 19.83 | 8.25E-06 |
| response to topologically incorrect protein (GO:0035966) | 73 | 12 | 0.68 | + | 17.74 | 5.73E-08 |
| chaperone-mediated protein folding (GO:0061077) | 62 | 9 | 0.57 | + | 15.67 | 5.20E-05 |
| ubiquitin-dependent ERAD pathway (GO:0030433) | 42 | 6 | 0.39 | + | 15.42 | 1.43E-02 |
| recognition of pollen (GO:0048544) | 48 | 6 | 0.44 | + | 13.49 | 2.87E-02 |
| cell recognition (GO:0008037) | 50 | 6 | 0.46 | + | 12.95 | 3.56E-02 |
| ERAD pathway (GO:0036503) | 53 | 6 | 0.49 | + | 12.22 | 4.82E-02 |
| response to oomycetes (GO:0002239) | 96 | 8 | 0.89 | + | 8.99 | 1.55E-02 |
| defense response to bacterium (GO:0042742) | 413 | 31 | 3.83 | + | 8.1 | 5.68E-15 |
| protein folding (GO:0006457) | 179 | 13 | 1.66 | + | 7.84 | 8.22E-05 |
| signal transduction (GO:0007165) | 1330 | 94 | 12.32 | + | 7.63 | 1.11E-51 |
| signaling (GO:0023052) | 1369 | 94 | 12.68 | + | 7.41 | 1.21E-50 |
| response to bacterium (GO:0009617) | 506 | 34 | 4.69 | + | 7.25 | 2.80E-15 |
| cell communication (GO:0007154) | 1618 | 100 | 14.99 | + | 6.67 | 1.60E-50 |
| response to heat (GO:0009408) | 226 | 13 | 2.09 | + | 6.21 | 1.05E-03 |
| protein phosphorylation (GO:0006468) | 1037 | 54 | 9.61 | + | 5.62 | 4.58E-21 |
| defense response to other organism (GO:0098542) | 805 | 41 | 7.46 | + | 5.5 | 7.19E-15 |
| phosphorylation (GO:0016310) | 1216 | 56 | 11.27 | + | 4.97 | 1.60E-19 |
| cellular response to stimulus (GO:0051716) | 2521 | 115 | 23.36 | + | 4.92 | 9.08E-47 |
| response to cadmium ion (GO:0046686) | 315 | 14 | 2.92 | + | 4.8 | 7.00E-03 |
| defense response (GO:0006952) | 952 | 42 | 8.82 | + | 4.76 | 3.56E-13 |
| response to external biotic stimulus (GO:0043207) | 1092 | 47 | 10.12 | + | 4.65 | 1.06E-14 |
| response to other organism (GO:0051707) | 1092 | 47 | 10.12 | + | 4.65 | 1.06E-14 |
| response to biotic stimulus (GO:0009607) | 1093 | 47 | 10.13 | + | 4.64 | 1.09E-14 |
| interspecies interaction between organisms (GO:0044419) | 1120 | 47 | 10.38 | + | 4.53 | 2.73E-14 |
| immune system process (GO:0002376) | 361 | 15 | 3.34 | + | 4.48 | 6.76E-03 |
| response to metal ion (GO:0010038) | 433 | 17 | 4.01 | + | 4.24 | 3.01E-03 |
| response to external stimulus (GO:0009605) | 1508 | 49 | 13.97 | + | 3.51 | 8.05E-11 |
| phosphate-containing compound metabolic process (GO:0006796) | 1845 | 56 | 17.09 | + | 3.28 | 1.32E-11 |
| cellular response to organic substance (GO:0071310) | 672 | 20 | 6.23 | + | 3.21 | 2.02E-02 |
| phosphorus metabolic process (GO:0006793) | 1890 | 56 | 17.51 | + | 3.2 | 3.55E-11 |
| response to stimulus (GO:0050896) | 5567 | 145 | 51.58 | + | 2.81 | 7.70E-34 |
| cellular response to stress (GO:0033554) | 1119 | 29 | 10.37 | + | 2.8 | 2.56E-03 |
| cellular response to chemical stimulus (GO:0070887) | 1088 | 28 | 10.08 | + | 2.78 | 4.56E-03 |
| regulation of cellular process (GO:0050794) | 4726 | 108 | 43.78 | + | 2.47 | 2.13E-17 |
| response to stress (GO:0006950) | 3090 | 69 | 28.63 | + | 2.41 | 1.38E-08 |
| protein modification process (GO:0036211) | 2496 | 55 | 23.12 | + | 2.38 | 6.16E-06 |
| cellular protein modification process (GO:0006464) | 2496 | 55 | 23.12 | + | 2.38 | 6.16E-06 |
| regulation of biological process (GO:0050789) | 5374 | 117 | 49.79 | + | 2.35 | 9.07E-18 |
| cellular protein metabolic process (GO:0044267) | 3370 | 72 | 31.22 | + | 2.31 | 2.67E-08 |
| protein metabolic process (GO:0019538) | 3539 | 75 | 32.79 | + | 2.29 | 1.10E-08 |
| macromolecule modification (GO:0043412) | 2813 | 57 | 26.06 | + | 2.19 | 5.31E-05 |
| biological regulation (GO:0065007) | 5956 | 120 | 55.18 | + | 2.17 | 1.15E-15 |
| organonitrogen compound metabolic process (GO:1901564) | 4613 | 77 | 42.74 | + | 1.8 | 3.66E-04 |
| cellular macromolecule metabolic process (GO:0044260) | 4404 | 72 | 40.8 | + | 1.76 | 2.37E-03 |
| cellular process (GO:0009987) | 11959 | 190 | 110.8 | + | 1.71 | 4.74E-20 |
| Unclassified (UNCLASSIFIED) | 5470 | 32 | 50.68 | - | 0.63 | 0.00E+00 |
| regulation of metabolic process (GO:0019222) | 3302 | 10 | 30.59 | - | 0.33 | 2.93E-02 |
| regulation of cellular metabolic process (GO:0031323) | 2956 | 8 | 27.39 | - | 0.29 | 3.99E-02 |
| regulation of macromolecule metabolic process (GO:0060255) | 3030 | 8 | 28.07 | - | 0.28 | 1.97E-02 |
| regulation of primary metabolic process (GO:0080090) | 2806 | 7 | 26 | - | 0.27 | 2.62E-02 |
| organic cyclic compound metabolic process (GO:1901360) | 2597 | 6 | 24.06 | - | 0.25 | 3.38E-02 |
| regulation of gene expression (GO:0010468) | 2648 | 5 | 24.53 | - | 0.2 | 4.77E-03 |
| regulation of RNA metabolic process (GO:0051252) | 2260 | 4 | 20.94 | - | 0.19 | 2.46E-02 |
| regulation of nucleobase-containing compound metabolic process (GO:0019219) | 2320 | 4 | 21.49 | - | 0.19 | 1.74E-02 |
| regulation of cellular macromolecule biosynthetic process (GO:2000112) | 2340 | 4 | 21.68 | - | 0.18 | 1.17E-02 |
| regulation of macromolecule biosynthetic process (GO:0010556) | 2353 | 4 | 21.8 | - | 0.18 | 1.18E-02 |
| regulation of cellular biosynthetic process (GO:0031326) | 2451 | 4 | 22.71 | - | 0.18 | 5.67E-03 |
| regulation of biosynthetic process (GO:0009889) | 2488 | 4 | 23.05 | - | 0.17 | 3.85E-03 |

Supplementary Table 11

(a) In a separate spreadsheet

ST11a - PAML analysis of D. cochinchinensis.xlsx

(b) In a separate spreadsheet

ST11b - PAML analysis of D. oliveri.xlsx

1. Russell, J. R. *et al.* tropiTree: An NGS-Based EST-SSR Resource for 24 Tropical Tree Species. *PLoS One* **9**, e102502 (2014).

2. Bertioli, D. J. *et al.* The genome sequences of Arachis duranensis and Arachis ipaensis, the diploid ancestors of cultivated peanut. *Nat. Genet.* **48**, 438–446 (2016).

3. Cannon, S. B. *et al.* Data from: Multiple polyploidy events in the early radiation of nodulating and non-nodulating legumes. *Dryad* (2015). Available at: https://doi.org/10.5061/dryad.ff1tq.

4. Griesmann, M. *et al.* Phylogenomics reveals multiple losses of nitrogen-fixing root nodule symbiosis. *Science* **361**, eaat1743 (2018).

5. Hane, J. K. *et al.* A comprehensive draft genome sequence for lupin (Lupinus angustifolius), an emerging health food: insights into plant-microbe interactions and legume evolution. *Plant Biotechnol. J.* **15**, 318–330 (2017).
